# DNA repair enzymes of the Antarctic Dry Valley metagenome

**DOI:** 10.3389/fmicb.2023.1156817

**Published:** 2023-04-14

**Authors:** Elizabeth Rzoska-Smith, Ronja Stelzer, Maria Monterio, Stephen C. Cary, Adele Williamson

**Affiliations:** ^1^Proteins and Microbes Laboratory, School of Science, University of Waikato, Hamilton, New Zealand; ^2^Thermophile Research Unit, School of Science, University of Waikato, Hamilton, New Zealand

**Keywords:** Antarctic Dry Valleys, metagenome, sequence similarity network, DNA repair, nuclease, DNA ligase

## Abstract

Microbiota inhabiting the Dry Valleys of Antarctica are subjected to multiple stressors that can damage deoxyribonucleic acid (DNA) such as desiccation, high ultraviolet light (UV) and multiple freeze-thaw cycles. To identify novel or highly-divergent DNA-processing enzymes that may enable effective DNA repair, we have sequenced metagenomes from 30 sample-sites which are part of the most extensive Antarctic biodiversity survey undertaken to date. We then used these to construct wide-ranging sequence similarity networks from protein-coding sequences and identified candidate genes involved in specialized repair processes including unique nucleases as well as a diverse range of adenosine triphosphate (ATP) -dependent DNA ligases implicated in stationary-phase DNA repair processes. In one of the first direct investigations of enzyme function from these unique samples, we have heterologously expressed and assayed a number of these enzymes, providing insight into the mechanisms that may enable resident microbes to survive these threats to their genomic integrity.

## Introduction

New areas of exploration and advances in detection methods during the last decade have revealed the diversity of life surviving in regions previously thought to be uninhabitable (Cary et al., 2010; Christner et al., 2014; Yung et al., 2014; Wei et al., 2016). One example are the McMurdo Dry Valley systems of Victoria Land Antarctica, the coldest and driest places on Earth with an environment that has been compared to the surface of Mars (Cary et al., 2010; Stomeo et al., 2012). Despite this, metagenomic surveys have demonstrated that the Dry Valleys support a low-complexity but diverse ecosystem which is dominated by bacteria and appears to be extremely reactive to change (Cary et al., 2010; Tiao et al., 2012; Yung et al., 2014; Chown et al., 2015; Wei et al., 2016; Lee et al., 2018, 2019; Niederberger et al., 2019). Abiotic stressors in this environment present particular challenges to DNA integrity, including multiple freeze–thaw cycles and desiccation leading to lethal double-stranded DNA breaks (Pitcher et al., 2007a; Stomeo et al., 2012), nutrient scarcity which limits DNA synthesis (Gourse and Keck, 2007) and UV radiation which induces base-dimer lesions (Goosen and Moolenaar, 2008).

Bacteria comprising environmental communities and inhabiting extreme environments are often slow-growing, adapted to low-nutrient conditions and include an enormous species diversity with many uncultured representatives (Stomeo et al., 2012; Christner et al., 2014; Le et al., 2016). In contrast, the model organisms on which our present understanding of DNA repair processes is based are predominantly pathogens and mesophiles from habitats of moderate temperature that are able to grow rapidly on rich media in pure culture (Aravind et al., 1999; Kreuzer, 2013). In these well-characterized bacterial repair systems, single-stranded DNA damage such as chemically modified bases, bulky UV-induced lesions and mismatches caused by replication errors are targeted by the excision-repair pathways that remove and replace the affected nucleotides, or by direct chemical reversal of the damage (Goosen and Moolenaar, 2008; Perugino et al., 2015; Brissett et al., 2020; Wozniak and Simmons, 2022). Double-strand breaks caused by collapsed replication forks, prolonged desiccation or ionizing radiation are repaired using RecA-dependent homologous recombination, which requires the presence of an intact complementary DNA copy (Kuzminov, 1999; Wang et al., 2022). Some species are also able to repair double-strand breaks directly *via* an error-prone non-homologous end-joining system that utilizes the Ku-dependent multifunctional ATP-dependent DNA ligase/polymerase/nuclease enzyme LigD (Bowater and Doherty, 2006; Shuman and Glickman, 2007).

Beyond these canonical mechanisms, studies of cultivatable DNA-damage resistant bacteria have identified novel clade-specific DNA repair systems. These include condensed nucleoid-dependent end-joining by the radiation-resistant extremophile *Deinococcus radiondurans*, which involves the *Deinococcus*-specific PprA DNA-binding protein and a dedicated DNA ligase (Blasius et al., 2007, 2008; Ishino and Narumi, 2015). In the last two decades, exponential growth of whole-genome sequences from cultivated extremophile microorganisms has provided an opportunity to take a sequence-driven approach to identify novel DNA-repair mechanisms. In one example, subsequent to the discovery of the LigD-dependent non-homologous end-joining pathway in bacteria, ATP-dependent DNA ligases have been annotated in the genomes of numerous bacteria in addition to their replicative NAD-dependent forms (Wilkinson et al., 2001; Williamson et al., 2016). Some of these, the LigC ligases, are involved in alternate base-excision pathways (Płociński et al., 2017) while for others their roles in specific repair pathways and possible interaction partners *in vivo* remain to be determined. In another recent example, a novel mismatch-specific endonuclease NucS was identified in *Mycobacteria* as well as species of Archaea, which appears to compensate for the lack of key proteins in the highly-conserved mismatch repair pathway (Castañeda-García et al., 2017; Ishino et al., 2018; Wozniak and Simmons, 2022).

Given the diversity of known mechanisms present in culturable, predominantly mesophilic, species investigated to date it is highly likely that divergent or entirely novel mechanisms operate in more taxonomically distant or niche-selected bacteria, such as Dry Valleys microbiota. DNA repair pathways operating at low temperature are particularly interesting, as psychrophilicity and psychrotolerance generally imply slow rates of replication and rationing of cellular resources (De Maayer et al., 2014; Nunn et al., 2014), meaning a second replicating chromosome may not be available to for homologous recombination-mediated repair (Kuzminov, 1999). Hyperthermophile-specific DNA modifying enzymes have been identified that are thought to be adaptations to DNA repair and protection at high temperatures (Perugino et al., 2009), and it is likely that specific mechanisms also exist for cold-tolerance.

The samples analyzed in this work were collected as part of the most comprehensive terrestrial survey of Antarctic bacterial ecology undertaken to date including extensive data on topographic features and physicochemical parameters in addition to biological composition (Lee et al., 2019; Bottos et al., 2020). These previous studies, which evaluated bacterial richness and community structure through sequencing ribosomal RNA, have shown that environmental factors such as salinity and moisture content are the main drivers influencing taxonomic richness and community composition; however biotic factors, in particular carbon fixation by cyanobacteria, remain essential determinants of ecosystem complexity (Lee et al., 2019; Bottos et al., 2020). In the present work we have undertaken further exploration of the genomic content of these samples by sequencing metagenomes from a sub-set of sites with a view to understanding adaptation to this harsh climate at the molecular level. Here we report the first detailed systematic exploration of DNA repair pathways from Antarctic metagenomes, and one of the first attempts at recombinant protein production from these unique samples.

## Methods

### Sample collection, DNA extraction and sequencing

Samples were collected from the Miers, Marshall, and Garwood valleys during the New Zealand Terrestrial Antarctic Biocomplexity Survey (nzTABS, https://ictar.aq/nztabs-science/) as part of a wide-ranging study into the influence of geochemistry, geology, climate, and biotic factors on the complexity of terrestrial ecosystems. Sample collection, geological characterization and environmental metadata of the sample site have been described previously (Lee et al., 2019; Bottos et al., 2020). Briefly, the site includes a predominantly ice-free area over 200 km^2^ with a mean annual temperature of −20°C and precipitation of less than 10 cm per year. Sample sites were selected as representatives of geographical tiles delineated by their topographic and geologic attributes and samples were collected over two successive austral summers (January 2009 and 2010). For DNA extraction, soil samples were resuspended in ultra-pure water or Tris HCl and DNA was extracted as described using either a modified Ctab method or *via* MoBio’s Power Soil DNA Isolation protocol (Qiagen; Lee et al., 2019; Bottos et al., 2020). Purified DNA was shipped to the Joint Genome Institute (JGI) for sequencing, subsequent to passing quality controls as recommended by guidelines provided on the JGI website.^1^ Sequencing was carried out according to the standard JGI metagenomic workflow. Briefly, whole genome shotgun sequences were sequenced from 300 bp paired-end libraries using an Illumina HiSeq 2,500 and reads were assembled using MEGAHIT v. 1.0.6 and annotated using the IMG Annotation Pipeline v.4. 13.0 (Markowitz et al., 2014). Final minimal drafts of 31 assembled metagenomes ranging in size from 46,280–5,011,667 genes (59,690,366 total) are publicly available through the JGI Genomes Online Database (GOLD). GOLD analysis project identifiers are given in the table in Supplementary 1 along with gene counts at each site for proteins discussed in detail here.

### Profile searching and retrieval

Raw Hidden Markov Model (HMM) profiles for Pfam searches were downloaded from the European Molecular Biology Laboratory (EMBL)’s Pfam site (http://pfam.xfam.org/, accessed May 2019). The list of Pfam domains used in this initial search is given in Table 1. Predicted protein-coding sequences from each Antarctic metagenome were downloaded from the JGI’s IMG/MER web portal as FASTA sequences^2^ and searched with the HMM profiles using the hmmsearch program from the HMMER suite 3.2.1.^3^ Sequences above the inclusion threshold (*E*-value <1) were downloaded in FASTA format. Where the Pfam family used in the initial search was known to be part of one or more multi-domain proteins, a second search was performed to create sub-sets with up to three additional domains (Table 2).

**Table 1:** Numbers of DV-metagenome sequences retrieved from HMM searches for each Pfam domain.

First search (catalytic domain)			Second search (appending domains)		
Family name	Pfam identifier (Interpro identifier)	# Sequences^a^	Family name	Pfam identifier (Interpro identifier)	# Sequences^b^
NucS	PF01939 (IPR002793)	8,119	-	-	-
Hjc	PF01870 (IPR002732)	2,819	-	-	-
Rad52_Rad22	PF04098 (IPR041247)	1,621	-	-	-
DdrB	PF12747 (IPR024305)	271	-	-	-
UvdE	PF03851 (IPR004601)	2,743	-	-	-
DNA_ligase_A_M	PF01068 (IPR012310)	23,640	-	-	20,044
			DNA_ligase_A_N	PF04675 (IPR012308)	1791
			LigD_N +/− PrimaseS	PF13298 +/− PF01896 (IPR014144 +/− IPR002755)	1805
DNA_Photolyase	PF00875 (IPR006050)	9,373	-	-	9,373
HhH-GPD	PF00730 (IPR003265)	24,895	-	-	23,450
			AlkA_N	PF06029 (IPR010316)	1,445
MutS_I	PF01624 (IPR007695)	3,947	-	-	1942
			MutS_II +/− MutS_II +/− I MutS_V	PF05188 +/− PF05192 +/− PF00488 (IPR007860 +/− IPR007696 +/− IPR000432)	2005

^a^Total number of sequences with the catalytic domain used in the first search; ^b^Number of sequences in each sub-group with a particular appending domain (where present).

**Table 2:** Nodes used in Sequence Similarity Network construction for different Pfam assignments.

Family names	Length threshold (aa)^a^	Total nodes in SSN^b^	# MG nodes^c^	# NCBI nodes (as %)^d^	Sequence identity (%)^e^	# Connected (Fragmentation %)
NucS	200	2094	1,578	516 (24.6)	28	366 (17)
Hjc	45	2,844	1976	868 (30.5)	26	427 (15)
Rad52_Rad22	150	1,340	665	675 (51.8)	26	75 (6)
Rad52_Rad22 (high ID threshold)					50	151 (11)
DdrB	80	302	210	92 (30.5)	25	151 (30)
UvdE	270	819	422	397 (48.5)	54	92 (21)
DNA_ligase_A_M	300	2,998	2,477	521 (17.4)	64	190 (7)
DNA_ligase_A_M + DNA_ligase_A_N	450	1,145	700	445 (38.9)	50	203 (7)
DNA_ligase_A_M + LigD_N +/− PrimaseS	500	1,005	650	355 (35.3)	54	78 (7)
DNA_Photolyase	160	6,556	3,933	2,623 (40.0)	40	679 (10)
HhH-GPD	110	25,680	18,321	7,359 (28.7)	25	69 (17)
HhH-GPD + AlkA_N	200	7,444	1,030	6,414 (86.2)	40	4,357 (3)
MutS_I	100	3,662	953	2,709 (74.0)	50	223 (11)
MutS_I + MutS_II +/− MutS_II +/− I MutS_V	200	4,510	2094	2,416 (53.6)	50	413 (21)

^a^Length cut-off applied to sequences before SSN constructuion; ^b^Number of nodes above the length threshold used in final SSN; ^c^Number of DV-metagenome nodes in the SSN; ^d^Number of NCBI nodes and their percentage in the SSN; ^c^Percentage identity threshold for edges retained in the SSN; ^d^Number of connected componenes (i.e., clusters or sinlge nodes) and percentage fragmentation calculated as total # nodes/# connected components.

As Pfam has now been merged with and assimilated into the Interpro database, equivalent Interpro identifiers are included in the table.

### Sequence similarity network construction and analysis

Sequence similarity networks were constructed for each set of sequences identified by hmmsearch using the EFI-EST server^4^ ‘Option C, Generate SSN from provided FASTA sequence’ (Zallot et al., 2019). To distinguish groups of sequences that were unique to the Antarctic metagenomes, a proportion of randomly-selected NCBI sequences was included in the SSN using the ‘Protein Family Addition Option’. A fraction value was set to give 25–35% NCBI sequences from the UniRef database in the total SSN, or, for particularly large families a fraction giving >500 UniRef sequences. The initial SSN used a permissive *E*-value of five for edge calculation, and alignment scores were set during the finalization step to give between 2 and 4 million edges. Minimum length thresholds were set based on the model length of the HMM profile used for the initial search, or in the case of multi-domain proteins, on the sum of the model lengths. No maximum length was set.

The full network for each SSN was downloaded for further processing in Cytoscape 3.8.2.^5^ The yFiles organic layout was applied to optimize node layout and visualize separate clusters, while sequences were colored by source as being of metagenome origin or from UniRef. The network was refined by progressively increasing the percentage similarity edge threshold until nodes resolved into distinct clusters. Refinement was considered complete when one of the following criteria was met: nodes had resolved into clusters where metagenomic sequences and UniRef sequences were separated; the edge threshold exceeded 50% identity. Sequence similarity networks where metagenome-only clusters were evident prior to these latter two conditions being met were considered to contain potentially novel metagenome DNA repair candidate genes and were analyzed further. SSNs where metagenome and UniRef sequences remained mixed to the point of network fragmentation or at higher edge values were considered unlikely to include unique metagenomic representatives.

### Selection of candidate genes for recombinant expression

Where defined clusters comprised primarily metagenome representatives, the sequences in these clusters were investigated further for their potential participation in novel or as-yet undescribed DNA repair pathways. FASTA sequences within metagenome clusters were first analyzed by hmmscan (HMMER suite 3.2.1; http://hmmer.org/) to exclude any spurious hits which were included due to the permissive threshold used in the initial hmmsearch. Sequences from unique clusters were then aligned using ClustLW 2.1 and partial sequences lacking probable start or stop codons or those that aligned poorly were discarded. For the three groups of protein selected for recombinant expression, the longest contig in the group was submitted to the PHASTER server (http://phaster.ca/) to detect genes that were part of bacteriophage rather than bacterial chromosomes (Arndt et al., 2016). From the shortlisted genes, priority for cloning was given to sequences from large contigs to enable future investigation of adjacent genes for related functions.

### Recombinant protein expression and purification

Clonal genes encoding proteins of interest were ordered from Twist Biosciences in the pTwist-ENTR vector with codon optimization for *Escherichia coli*. Ordered constructs included an N-terminal His-tag and cleavage recognition site for the tobacco mosaic virus protease (TEV-protease) for tag removal. Genes were sub-cloned into the expression vectors pDEST17 (Invitrogen) and pHMGWA (GenBank #Eu680841) using the Gateway™ LR reaction kit (Thermofisher) according to the manufacturer’s instructions. Resulting expression constructs were transformed into chemically-competent DH5α cells for propagation.

To generate truncated DV-1-1-lig and DV-1-1-nuc genes, insert *att* sites for gateway cloning and to add the TEV cleavage site to the DV-1-1-lig product, a two-step PCR protocol was used with primer sequences given in Supplementary 2. In the first round the pDONR221 plasmid containing the DV-1-1-Nuc-Lig gene insert was used as a template to amplify both DV-1-1-Nuc segment (primer pair DV1-1Nuc Forward and DV1-1Nuc Back) and the DV-1-1-Lig segment (primer pair DV1-1Lig Forward and DV1-1Lig Back). PCR products were visualized on a 1% agarose gel and bands, corresponding to the correct PCR product size (1.73 bp for DV-1-Lig and 1.248 bp for DV-1-1-Nuc), were excised and extracted using the QIAquick ®, Gel extraction kit (QIAGEN). The second PCR step added *att*-sites to DV-1-1-Nuc (primer pair DV1-1 Forward-2, DV1-1Nuc Back) and DV-1-1-Lig (primer pair DV1-1 Forward-2, DV1-1Lig Back) generating products suitable for Gateway cloning (1.765 bp for DV-1-Lig and 1.279 bp for DV-1-1Nuc). The PCR products were cloned into the Gateway donor vector, pDONR221 (Invitrogen), using the using the Gateway™ BP reaction kit (Thermofisher) and transformed into chemically-competent DH5α cells, then further sub-cloned into the expression vector pDEST17 as described above.

To determine optimal production conditions, expression from each plasmid was tested in the *E. coli* expression strains BL21(DE3)-pLysS (Novagen), Origami (DE3; Novagen) and ArcticExpress (DE3; Aligent) at 15, 18, and 20°C. For small-scale expression trials, 50 mL cultures of cells were grown in conical flasks on Terrific Broth, 50 μg/mL ampicillin at 37°C with shaking at 180 rpm. Upon reaching an OD_600_ between 0.3 and 0.4 the temperature was adjusted and cells were equilibrated for 30 min before addition of 0.5 mM IPTG to induce expression. Cells were harvested after 18 h by centrifugation, resuspended in 2 mL lysis buffer (50 mM Tris pH 8.0, 750 mM NaCl, 1 mM MgCl_2_, 5% v/v glycerol) and lysed by sonication on ice. Insoluble material was pelleted by centrifugation and the soluble fraction was incubated with 20 μL of nickel beads (Cytiva) for 15 min and recovered by centrifugation followed by two washes with lysis buffer. Protein-bound nickel beads, insoluble fractions and soluble fractions were electrophoresed on 12% SDS-PAGE gels with successful expression being indicated by a strong band at the expected molecular weight in the nickel-bead fraction and, in some cases, the soluble sample. Expression as inclusion bodies was indicated as a band at this molecular weight in the insoluble sample.

Optimal conditions (listed in Supplementary 3) were used for large-scale cultivation for purification. Cultures (1–4 L) were grown at the appropriate temperatures as identified from the small-scale cultures and harvested by centrifugation. Cells were lysed by sonication in lysis buffer and clarified by centrifugation and passage through a 0.45 μm filter. Protein purification was carried out at 4°C on an Äkta Prime FPLC as described previously (Williamson and Pedersen, 2014). Briefly, clarified lysate with an additional 10 mM imidazole was purified by immobilized affinity chromatography (IMAC) using 5 mL His-Trap HP column (Cytiva) and washed with 5–10 column volumes of buffer A (50 mM Tris pH 8.0, 750 mM NaCl, 5% v/v glycerol, 10 mM imidazole). His-tagged proteins were eluted with Buffer B (50 mM Tris pH 8.0, 750 mM NaCl, 5% v/v glycerol, 500 mM imidazole) on a gradient from 0 to 100% over 75 mL. For tag removal, fractions containing the target protein were exchanged into Buffer C (50 mM Tris pH 8.0, 100 mM NaCl, 1 mM DTT, 5% v/v glycerol) using a HiPrep 26/10 Desalting column (Cytiva) and incubated at 4°C overnight with 1 mg of TEV-protease which had been produced in-house according to published protocols (Tropea et al., 2009). Cleaved proteins were subjected to reverse IMAC by re-application to the His-Trap column. To increase purity and remove high molecular-weight aggregates, the flow-through which contained the untagged target protein was up-concentrated to less than 5 mL volume and loaded onto a either a Superdex200 16/600 (S200) or Superdex75 16/600 (S75) column. In cases where the tag was not removed by TEV cleavage, fractions from the initial gradient elution in Buffer B were pooled and used directly this gel-filtration polishing step. Final purified protein was up concentrated to 0.5–5 mg mL^−1^, mixed 50:50 v/v with glycerol and stored at −80°C.

### Assay of DNA binding and nuclease activity

Enzymatic activity on DNA-damage substrates (Supplementary 4B), flapped/splayed substrates (Supplementary 4C) and DNA ligase (Supplementary 4D) were analyzed by denaturing gel electrophoresis, while nuclease substrates (Supplementary 5B) and substrate binding experiments were analyzed by native PAGE. Substrates were generated from oligonucleotides (IDT) with synthetically-incorporated DNA damages and fluorescent labels which are listed in Supplementary 6. These were annealed in the combinations given in Supplementary 7 as described previously (Sharma et al., 2020) using final concentrations of 80 nM for the 5′ FAM-labeled probe strand and 112 nM for the unlabeled strand (damage, nuclease and double-strand break ligase substrates) or 400 nM unlabeled strands (nicked ligase substrates). All assays were carried out in 50 mM Tris pH 8.0, 50 mM NaCl, 10 mM DTT. Enzyme activity assays also included 10 mM Mg^2+^ or Mn^2+^ as specified, while for binding experiments this was replaced with 5 mM EDTA. For DNA ligase assays, 1 mM of nucleotide cofactor (ATP, ADP, or NAD^+^) was used as specified. Reactions were initiated by addition of protein and were incubated at the temperatures and times indicated. For the DNA ligase nucleotide preference experiments, enzyme was pre-incubated with unlabeled DNA for 2 h at 20°C to ensure any enzyme purified in the pre-adenylated state was turned over. After this time, labeled DNA substrate and 1 μM of cofactor (ATP, ADP, or NAD) was added to the reaction and incubated for indicated times.

Activity assays for damage, flapped/splayed and ligase substrates were quenched by addition of Quench Buffer to give a final concentration of 25% formamide, 20 mM EDTA, 0.05% bromophenol blue and heated to 95°C for 5 min before electrophoresis on 20% denaturing TBE urea gels (20% acrylamide/Bis-acrylamide 29:1, 7 M urea, 1x TBE). For binding and nuclease assays Loading buffer was added to give a final concentration of 20 mM EDTA, 0.05% bromophenol blue, 5% glycerol before the samples were electrophoresed on a 10% TBE gel (10% acrylamide/Bis-acrylamide 29:1, 1x TBE). Gels were visualized on an iBright imager (Invitrogen) using the fluorescein setting.

### Differential scanning fluorimetry

Thermal stability measurements of DV-Hjc were carried out by differential scanning fluorimetry (DSF) as previously described (Ericsson et al., 2006). The pH was adjusted by dilution into Britton-Robinson universal buffers over a rage of 5.0–9.5 to give a final DV-Hjc concentration of 0.2 mg/mL and final SYPRO dye concentration of 1.2×. Unfolding over a temperature range from 25 to 98°C was measured using a RotoGene Q thermocycler (Qiagen) and the *T*_m_ was determined by plotting the first derivative of the melt data.

## Results and discussion

### Dry Valley metagenomes contain numerous DNA replication and repair proteins

**Figure 1:**
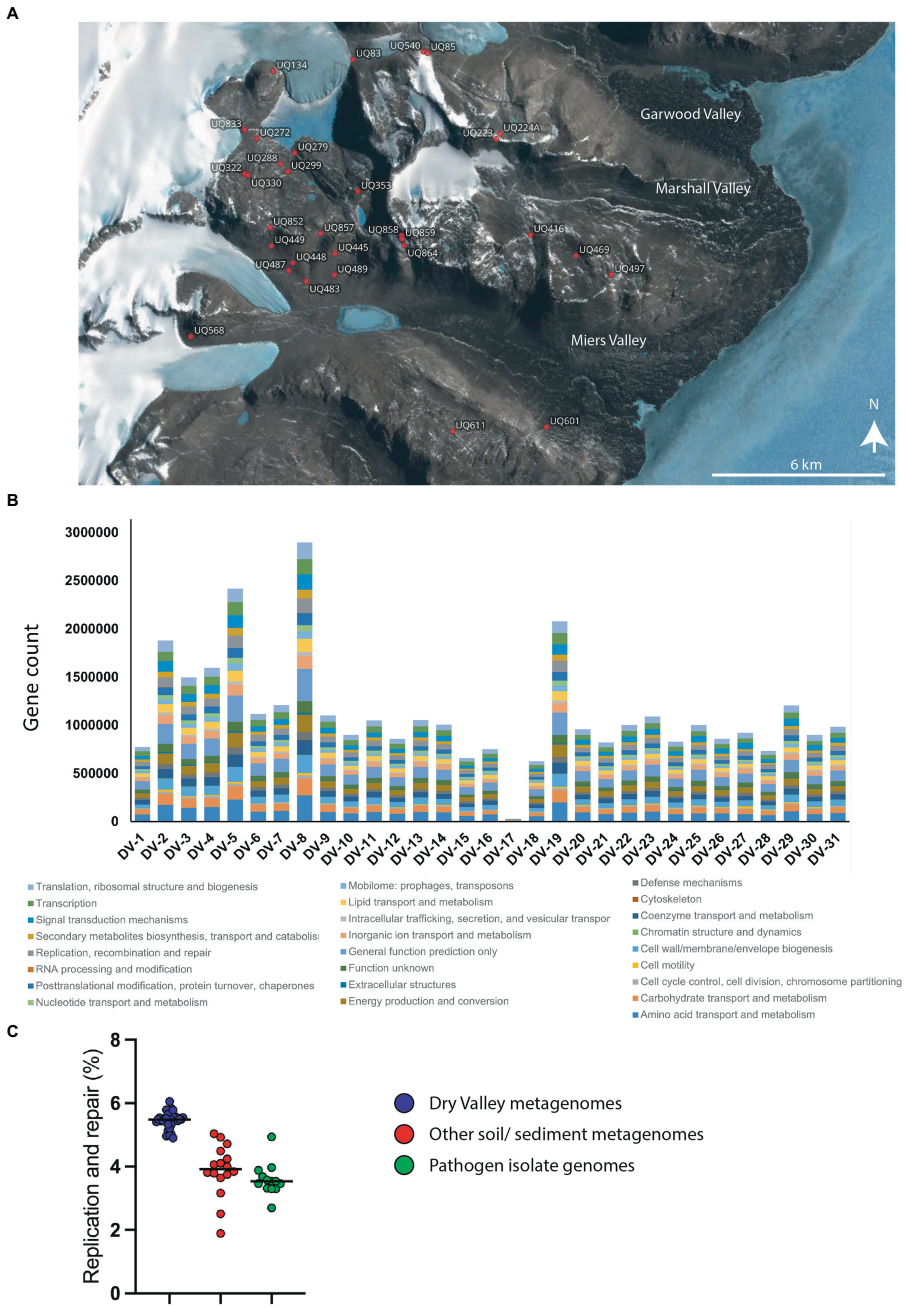
**(A)** Map of sample sites for metagenome sequences. The location of each sample was plotted using Quantarctica (v3.1) (Matsuoka et al., 2021). **(B)** Counts of genes in COG categories for metagenome sequences from each site. **(C)** Percentage of genes in the COG category ‘DNA replication and repair’ from Dry Valley metagenomes (blue), other soil/ sediment metagenomes (red) or genomes from isolated pathogens (green). Values for datapoints are a percentage of the total genes in COG categories for each sample and are given in Supplementary 1 together with GOLD identifiers and sample type. Numbers and percentages of genes in all COG categories for all samples are shown in Supplementary 9, 10, respectively.

Of the almost 60 million genes predicted in the 30 sequenced metagenomes from Dry Valleys, 58% (34,784,791 genes) were assigned to Clusters of Orthologous Groups (COG) categories (Figures 1A,B). Consistent with our hypothesis that these microbes will possess effective or specialized DNA repair systems to cope with environmental stressors, 5.5% of these annotated Dry Valley metagenome genes belonged to the ‘Replication, Recombination and Repair’ COG category (Figure 1C). This is higher than the proportion found in other environmental metagenomes including forest soils (3.9%), other soil communities (3.6%) and freshwater sediments (4.3%). It is also considerably higher than the proportion of DNA repair genes found the genomes of isolates of human pathogens which are the model organisms on which most of our understanding of bacteria DNA repair is based (Figure 1C).

To further explore whether these sequences represent novel or divergent DNA repair enzymes, translated predicted coding genes were searched for distant homology to known DNA repair proteins using hmmsearch. Probe domains used in the first hmmsearch were chosen on the basis that (i) they comprise a large family with many representatives involved in DNA repair, or (ii) they represent a recently-described or specialist DNA repair mechanism (Pavlopoulou et al., 2016; Lilley, 2017; Wozniak and Simmons, 2022). This included single-domain proteins of the NucS/ EndoMS endonucleases (NucS; Castañeda-García et al., 2017; Ishino et al., 2018), Holliday junction resolvase-type endonucleases (Hjc; Lestini et al., 2010; Huang et al., 2015), UV-damage excision endonucleases (UvdE; Lim et al., 2019), light-activated photolyase proteins for direct reversal of UV-induced pyrimidine-pyrimidine dimers (DNA_photolyase; Yi and He, 2013), *Deinococcus* radiation resistance proteins DdrA (Rad52_Rad22) and DdrB (DdrB; Lim et al., 2019), as well as multi-domain families such as ATP-dependent DNA ligases (DNA_ligase_A_M), excision-repair glycosylases (HhH_GPD) and mismatch-recognition proteins (MutS_I; Bruner et al., 2000; Otani et al., 2013; Pidugu et al., 2021). The former single-domain proteins were searched with a primary catalytic HMM profile only, while the latter multi-domain proteins were sub-divided by a second set of search criteria using up to three additional HMM domain searches. Hits were retrieved for all primary domains and sub-domains used in this search, indicating that DV-metagenomes possess a diverse array of DNA repair enzymes (Table 1). In particular, large numbers of NucS homologs (>8,000) and ATP-dependent DNA ligases (>23,000) were identified which is notable as neither pathway is ubiquitous among bacteria and both are absent from many standard mesophilic model organisms such as *E. coli* (Wilkinson et al., 2001; Creze et al., 2011; Williamson and Leiros, 2020; Zhang et al. 2020a,b). Sequences above the inclusion threshold (E-value <1) were downloaded and used to build SSNs. To minimize inclusion of partial or small fragments of proteins, length thresholds corresponding to minimum domains lengths of the search model were applied during SSN construction. For some families, this decreased the proportion of sequences remaining in the final SSN considerably, for example <20% of Nuc S and < 15% of DNA ligase DNA_ligase_A_M-only proteins were retained (Table 2). The final SSNs also included a proportion of NCBI sequences retrieved from UniRef which served as outgroups allowing clusters of DV-metagenome sequences with unique features to be distinguished (Table 2). The target proportion of NCBI sequences was 30–50% of the total nodes in the SSN; however, for functionally-diverse families with a large number of representatives such as proteins containing AlkA and MutS_I domains, the number was increased to ensure that the range of sequence diversity within the family was accurately reflected. Conversely, the application of the length threshold in the DNA_ligase_A_M dataset meant a large number of NCBI sequences were excluded and the final SSN included fewer than 25%.

Full SSNs with permissive (low) edge threshold values were downloaded and manually refined until either distinct DV metagenome clusters resolved, or until the percentage threshold retained exceeded 50% sequence identity. An approximate measure of SSN cohesiveness was determined by the percentage fragmentation, calculated as the proportion of connected components (clusters or single nodes) relative to the total number of nodes. This remained under 20% for all SSNs except DdrB (30%), UvsE and MutS_I +/-II +/-III +/-V (both 21%). Five domain families resolved unique DV-metagenome clusters at edge thresholds below 30%; NucS, Hjc, Rad52_Rad22, DdrB and HhH-GPD, suggesting that some of these sequences may encode specialized adaptations. Four additional families, DNA_photolyase, UvsE and the DNA ligase groups DNA_ligase_A_M + A_N and DNA_ligase_A_M + LigD_N + primase resolved unique clusters before or just above 50% sequence identity. In contrast the DNA ligase DNA_ligase_A_M network was refined to more than 60% before resolution of predominantly metagenome clusters. No significant clusters of unique sequences were resolved for the MutS_I sequences at the 50% threshold, either with or without additional domain searches, and this family was not investigated further (Supplementary 11).

### Dry Valley metagenomes encode unique groups of endonuclease proteins

**Figure 2:**
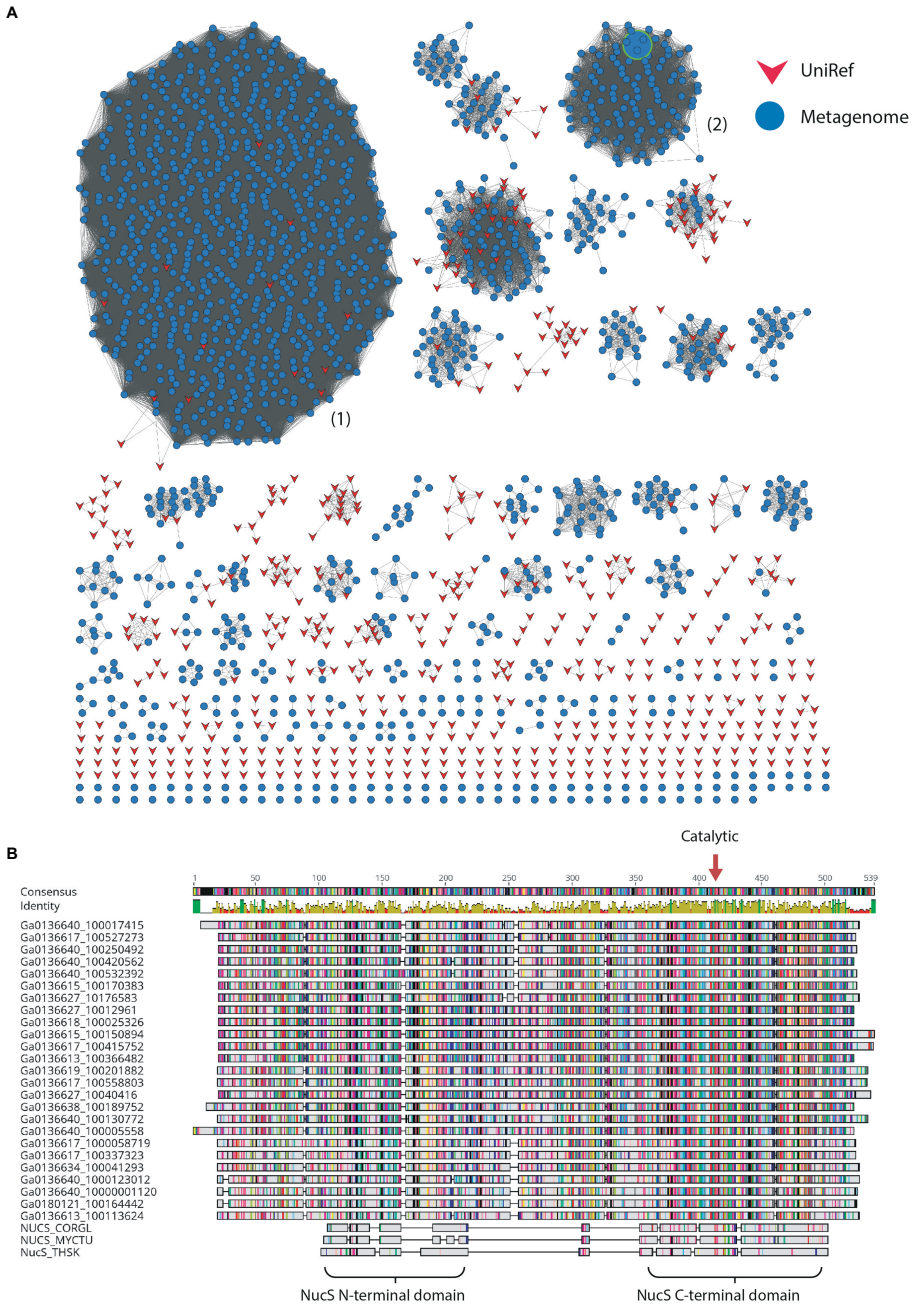
**(A)** Sequence Similarity Network for NucS-type proteins at 28% identity threshold; other network parameters are detailed in Table 2. Dry-Valley metagenome nodes are colored blue, UniRef50 nodes are indicated in red. Cluster numbers discussed in the text are given in parentheses. The node corresponding to the recombinantly produced homolog from Cluster (2) is indicated with a large green-bordered symbol. **(B)** Alignment of full-length sequences from Cluster (2) with characterized NucS/EndoMS from *Corynebacterium glutamicum* (NUCS_CORGL), Mycobacterium tuberculosis (NUCS_MYCTU) and *Thermococcus kodakarensis* (NUCS_THSK). Domains identified in the crystal structure of *T. kodakarensis* NucS/EndoMS are indicated below the alignment and the position of the conserved catalytic aspartic acid residue is indicated with a red arrow.

**Figure 3:**
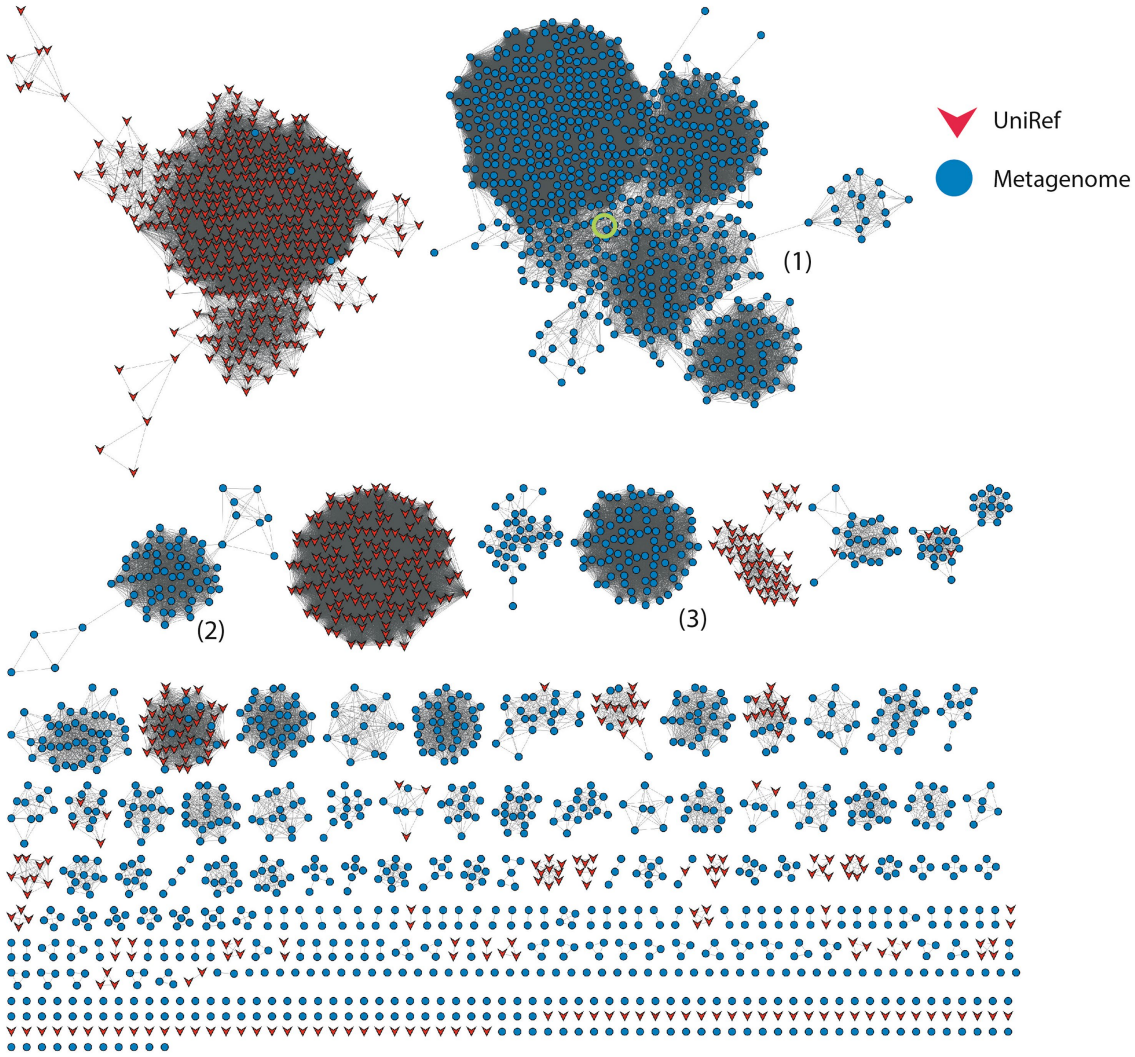
Sequence Similarity Network for Hjc-type proteins at 26% identity threshold; other network parameters are detailed in Table 2. Dry-Valley metagenome nodes are colored blue, UniRef90 nodes are indicated in red. Cluster numbers discussed in the text are given in parentheses. The node corresponding to the recombinantly produced homolog from Cluster #1 is indicated by the green circle.

**Figure 4:**
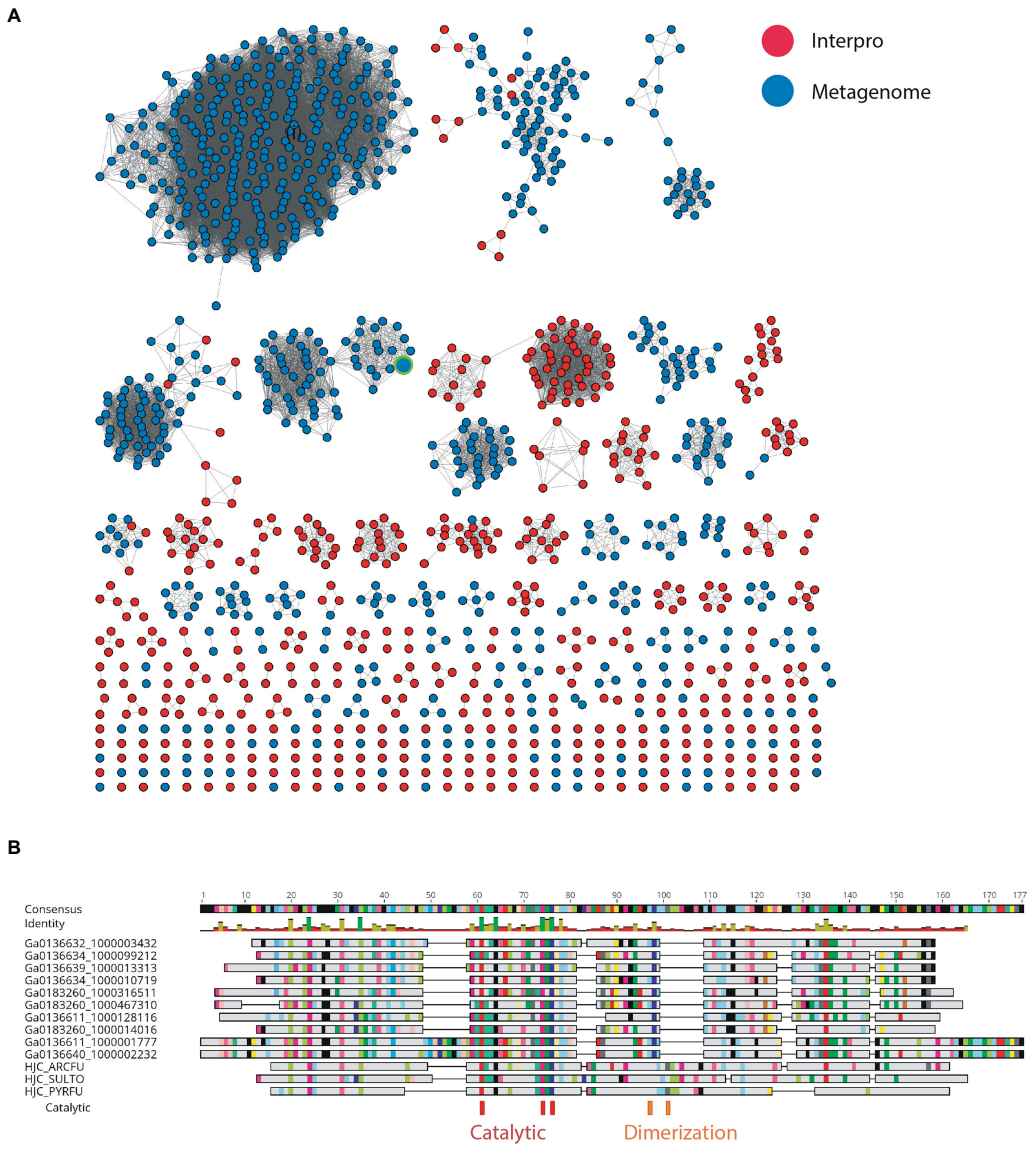
**(A)** SSN of metagenome hits to UPF0102 and Pfam members. The node corresponding to the recombinantly produced homolog in is indicated by the green border. **(B)** Alignment of selected full-length sequences from metagenome to characterized Hjc homologs from *Archeoglobus* fulgidus (HJC_ARCFU), *Pyrococcus furiosus (HJC_PYRFU) and* Sulfurisphaera tokodaii str. 7 (HJC_SULTO).

Nuclease enzymes are ubiquitous participants in almost all known DNA repair pathways with endonucleases playing a key role in excision repair pathways that remove damaged or mis-matched nucleotides within DNA duplexes (Wozniak and Simmons, 2022). The NucS/EndoMS are a recently-discovered family of endonucleases that carry out an alternative mismatch repair pathway in Archaea and Bacteria that lack the canonical MutS-MutL-based system (Ishino et al., 2016;Castañeda-García et al., 2017; Ishino et al., 2018). A large number of sequences with matches to the NucS (PF01939) profile were identified in the DV metagenome, and two major DV-metagenome-containing clusters were resolved at 28% edge identity each with 761 and 115 metagenomic sequences, respectively (Figure 2A). No appending domains were detected by hmmscan for sequences in either cluster, and for both the most significant homology is to the Endonuclease NucS family (Table 3). Interestingly, neither cluster was reliably annotated as NucS/EndoMS by the IMG pipeline (only 3 of the 761 Cluster (1) sequences) indicating these homologs would not have been detected by searching IMG alone. Cluster (1) includes 12 nodes from the UniRef50 family which comprise representative nodes for sequences with >50% identity in UniRef. The largest UniRef50 node represents 2,150 individual sequences from diverse phyla (Acidobacteria, Actinobacteria, Bacteroidetes, Chlamydiae, Firmicutes, Gemmatimonadetes, Proteobacteria and Rhodothermaeota) while the second largest node represents 794 individual sequences from Actinobacteria and Chlamydiae. This suggests that although the number of UniRef50 nodes included is small, this cluster in fact represents the majority of bacterial NucS sequences including *Mycobacterium tuberculosis* and *Corynebacterium glutamicum* both of which have been characterized in the past (Castañeda-García et al., 2017; Ishino et al., 2018). Cluster (2) by contrast includes only DV-metagenome sequences some of which are more than 500 amino acids in length, which is considerably longer than characterized homologs. Alignment of 25 full-length Cluster (2) sequences with sequences of characterized NucS homologs from *C. glutamicum*, *M. tuberculosis* and *Thermococcus kodakarensis* indicates that the DV metagenome sequences are extended N-terminally relative to the characterized NucS homologs (Figure 2B). The best aligned portion are the final 200 C-terminal residues which correspond to the RecB-like endonuclease domain of the structurally-characterized NucS/EndoMS of *T. kodakarensis*, while there is poor alignment with the N-terminal domain which is involved in mismatch recognition and DNA binding. The 100-residue extension is highly conserved among DV-metagenome NucS homologs and does not have any counterpart in the characterized sequences. The NucS proteins of bacteria provide one recent example where previously-undetected homologs of extremophile archaeal proteins are involved in DNA-damage resistance in bacterial species. Other examples are the LigC DNA ligases and Prim-pol polymerases which participate in an alternate stationary-phase base-excision repair in some bacterial species (Płociński et al., 2017; Brissett et al., 2020). Based on this observation, we considered whether homologs of other Archaeal-type DNA repair proteins could play a role in survival by DV species of bacteria. The archaeal Holliday junction resolvase (Hjc) family includes enzymes with an equivalent function to the bacterial RuvC endonuclease that cleaves Holliday junctions formed during homologous recombination (Shin et al., 2014; Wyatt and West, 2014). These archaeal resolvases possess a restriction endonuclease-type fold which is distinct from the RuvC and bacteriphageT4-endoVII resolvase structures (Nishino et al., 2001; Wyatt and West, 2014; Lilley, 2017). Hmmsearch identified almost 3,000 hits to the Hjc domain in the Dry-Valleys dataset, and SSN analysis of these sequences showed they form separate clusters from UniRef at 26% identity with three of the six clusters containing exclusively DV-metagenome sequences (Figure 3A). Hmmscan indicates that clusters 1 and 2 both have hits to unknown protein domain UPF0102 and no highly significant hits to other domains with known enzymatic functions although there are low-probability hits to both Hjc and nuclease family enzymes (Table 3). Cluster 3 has high probability hits to elongation domain (GTP_EFTU) and is therefore discounted from further analysis. The UPF0102 family is present in a wide variety of bacterial taxa, and as these were not included as Uniref representatives in the Hjc SSN, a new network was built using the 484 reviewed UPF0102 sequences in Interpro. Metagenome-only clusters separated out at threshold of 64% identity (Figure 3B). Several of the Interpro sequences are annotated as the transcription factor YraN, for example the members of the most significant Interpro sequence cluster which contains Enterobacteriaceae representatives (Figure 3B, Cluster (i)). However, neither the Interpro nor the DV-metagenome sequences align well to the characterized YraN protein from *Bacillus subtilis*, nor to known restriction enzymes (data not shown). Alignment of a selection of full-length sequences to the archaeal Holliday junction resolvases shows that the three key catalytic residues required for nucleolytic activity in *Pyrococcus furiosus* Hjc, Asp 33, Glu 46, Lys 48 are fully or functionally conserved; however other positions important for catalysis (Glu 9, Arg 10 and Arg 25) and dimer formation (Val 24, Val35, Phe 68 and Phe 72) are not (Figure 3C; Komori et al., 2000; Nishino et al., 2001). Although our analysis has not identified any close bacterial equivalents of the archaeal Hjc Holliday junction resolvases in Dry-Valley bacteria, a large number of more distantly-related UPF0102 were found in these metagenomes and are worthy of further investigation (Figure 4).

**Table 3:** Analysis of sequence similarity network clusters from Dry-Valley metagenome sequences.

Catalytic domain^a^	Other domains^b^	Cluster (DV sequence count)	Top hmmscan hit^c^ (E-value)	Annotation from IMG
NucS	-	1 (761)	Endonuclease NucS (7.60E-93)	Hypothetical protein		2 (115)	Endonuclease NucS (3.50E-06)	Hypothetical protein
Hjc	-	1 (722)	Uncharacterised UPF0102 (5.10E-30)	Putative endonuclease		2 (71)	Uncharacterised UPF0102 (1.60E-29)	Putative endonuclease		3 (88)	GTP_EFTU (7.00E-56)	GTP-binding protein
Rad52_Rad22	-	1 (227)	Rad52_Rad22 (1.90E-18)	Hypothetical protein		2 (303)	Rad52_Rad22 (3.90E-12)	Hypothetical protein
DdrB	-	1 (9)	DdrB (7.20E-60)	DdrB-like protein		2 (8)	DdrB (1.20E-14)	DdrB-like protein		3 (17)	AICARFT_IMPCHas (1.00E-113)	Phosphoribosylaminoimidazolecarboxamide formyltransferase / IMP cyclohydrolase		4 (15)	GDPD (1.90E-13)	Glycerophosphoryl diester phosphodiesterase		5 (9)	DdrB (0.034)	Hypothetical protein		6 (7)	GATase_6 (5.10E-30)	Asparagine synthase (glutamine-hydrolysing)		7 (9)	DUF150 (1.10E-20)	Ribosome maturation factor RimP
UvdE	-	1 (146)	UvdE (5.40E-60)	UV DNA damage endonuclease		2 (98)	UvdE (3.20E-70)	UV DNA damage endonuclease		3 (80)	UvdE (2.20E-53)	UV DNA damage endonuclease
DNA_ligase_A_M	-	1 (345)	DNA_ligase_A_M (2.00E-39)	Non-homologous end joining protein LigD		2 (141)	DNA_ligase_A_M (4.30E-38)	Non-homologous end joining protein LigD		3 (70)	DNA_ligase_A_M (1.30E-34)	Non-homologous end joining protein LigD		4 (47)	SecA_DEAD (2.30E-130)	Preprotein translocase subunit SecA	DNA_ligase_A_N	1 (145)	DNA_ligase_A_M (3.70E-47)	DNA ligase I	LigD_N +/− PrimaseS	1 (118)	LigD_N (5.60E-37)	Non-homologous end joining protein LigD	2 (34)	DNA_ligase_A_M (3.20E-37)	Non-homologous end joining protein LigD	3 (23)	LigD_N (3.40E-38)	Non-homologous end joining protein LigD	4 (24)	LigD_N (1.30E-38)	Non-homologous end joining protein LigD	5 (14)	LigD_N (7.10E-34)	Non-homologous end joining protein LigD
DNA_Photolyase		1 (124)	E1-E2_ATPase (7.1E-58)	Cu2 + −exporting ATPase		2 (80)	HAD_2 (9.20E-14)	Uncharacterized protein		3 (46)	SIR2 (6.6E-45)	NAD-dependent deacetylase
HhH-GPD	-	1 (1766)	HhH-GPD (1.20E-17)	Endonuclease-3		2 (1137)	PD40 (4.90E-38)	DNA-3-methyladenine glycosylase II		3 (458)	HhH-GPD (2.30E-09)	N-glycosylase/DNA lyase		4 (1500)	HhH-GPD (5.90E-18)	HhH-GPD superfamily base excision DNA repair protein		5 (488)	HhH-GPD (2.80E-08)	3-methyladenine DNA glycosylase/8-oxoguanine DNA glycosylase		6 (507)	HhH-GPD (6.50E-18)	Base-excision DNA repair protein		7 (673)	HhH-GPD (3.00E-13)	DNA-3-methyladenine glycosylase II/AraC family transcriptional regulator		8 (382)	Adenine_glyco (1.40E-83)	DNA-3-methyladenine glycosylase I	AlkA_N	1 (912)	AlkA_N (2.30E-40)	DNA-3-methyladenine glycosylase II/AraC family transcriptional regulator		2 (54)	HhH-GPD (4.60E-32)	DNA-3-methyladenine glycosylase II/AraC family transcriptional regulator

a First search.

b Second search.

c Pfam name.

### Dry Valley metagenomes encode numerous homologs of *Deinococcus* repair proteins

The UvdE endonucleases are involved in the repair of UV-induced cyclobutane pyrimidine dimers (CPD) and 6–4 pyrimidine pyrimidones (6-4PP) and induced bipyrimidine photoadducts as well as oxidative damages. This protein is found in all radiation-resistant *Deinococcus* species sequenced to date, where it functions as a back-up to the nucleotide excision repair pathway (Earl et al., 2002; Timmins and Moe, 2016). Of the more-than 400 DV-metagenome sequences retrieved by searching with the UvdE HMM, 324 formed three distinct predominantly DV-metagenome clusters at 54% identity, all of which are predicted to be UvdE proteins by both hmmscan and IMG annotation (Table 3 and Supplementary 12A). A number of DV-metagenome sequences also group with the smaller UniRef sub-cluster which contains characterized bacterial UvdE sequences. Alignment of full-length representatives of all three clusters shows that despite low sequence identity, none possess any remarkable truncations or extensions relative to *Deinococcus radiodurans* UvsE (Supplementary 12B).

**Figure 5:**
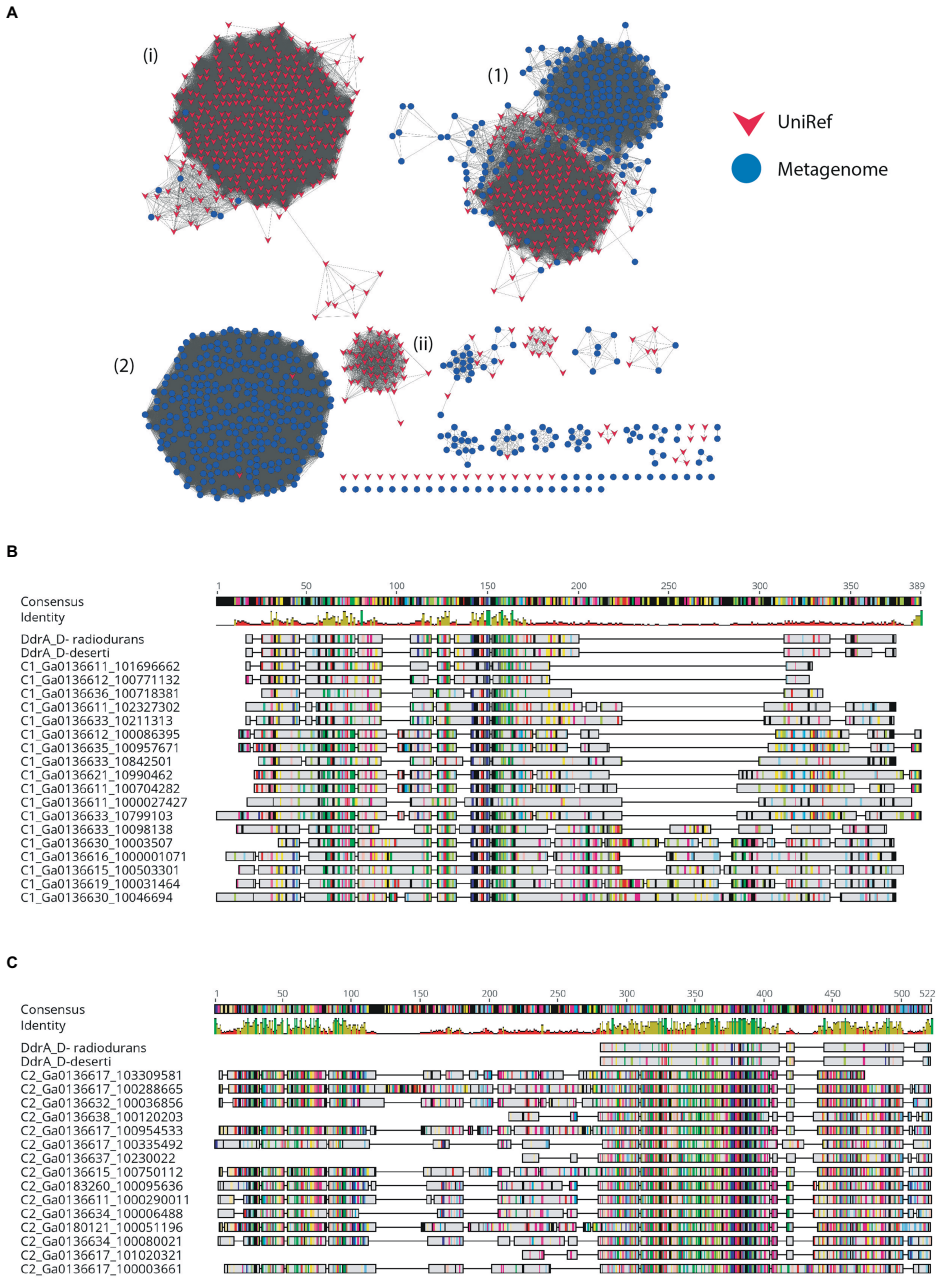
SSN of metagenome hits to the Rad52/Rad22 family. **(A)** SSN with 26% identity edge threshold. Dry-Valley metagenome nodes are colored blue, UniRef50 nodes are indicated in red. Cluster numbers discussed in the text are given in parentheses. **(B)** Alignment of selected full-length Cluster #1 proteins with DdrA from *Deinococcus radiondurans* and *Deinococcus deserti*. **(C)** Alignment of selected full-length Cluster #2 proteins with DdrA from *D. radiondurans* and *D. deserti*.

The Rad52/Rad22 family are single-strand annealing proteins and include the DNA Damage Response A (DdrA) single-strand annealing protein which is involved in radiation resistance by species of *Deinococcus*, as well as the Rad52 homologous recombination protein of Eukaryotes. DdrA proteins are specific to *Deinococcus* genomes and function to bind and protect single-stranded DNA ends, likely by self-associating to form heptameric rings which then form higher-order assemblies with single-stranded DNA (Harris et al., 2004; Gutsche et al., 2008; Selvam et al., 2013). In yeast, Rad52 increases the rate of assembly of the Rad51-single-strand DNA filament during homologous recombination and in humans in functions to promote strand annealing (Kowalczykowski, 2015). The 665 DV-metagenome hits to the Rad52_Rad22 HMM identified by hmmsearch formed two major clusters at a 26% identity threshold, with all sequences in both clusters annotated as hypothetical by IMG (Table 3). The first cluster of 227 DV-metagenome sequences (Figure 5A) (1) is merged with 201 UniRef sequences, with these separating from each other at a 50% identity level (Supplementary 13A,B). The majority (96%) of these UniRef sequences are also bacterial and include the DdrA protein from *D. radiodurans*. Two additional predominantly-UniRef clusters at the 26% identity level (Figure 5A) (i) and (ii) are almost exclusively eukaryotic and include the Rad52 DNA repair proteins. The second major metagenome cluster (Figure 5A) (2) comprises 303 DV-metagenomic sequences and only two UniRef representatives, both of which are from Acidobacteria. Alignment of a representative selection of full-length metagenome sequences with two DdrA sequences from *D. radiodurans* showed that many of these DV-metagenome sequences were significantly longer than DdrA. Several Cluster #1 DV-metagenome sequences include an insertion of about 100 bp in the C-terminal region (Figure 5B), while the Cluster #2 sequences have a large N-terminal extension which has no counterpart in DdrA (Figure 5C).

DdrB, together with DdrA is induced in the genome of *D. radiodurans* during radiation damage (Norais et al., 2009). It is a single-strand binding protein implicated in mediating double-strand break repair *via* a RecA-independent single-strand annealing mechanism (Norais et al., 2009). Of the 271 sequences retrieved by searches with the DdrB HMM, the majority of clusters at a 26% edge threshold [48 sequences in clusters (3), (4), (6), and (7)] were identified as having non-DNA repair functions by hmmscan (Table 3). The 17 sequences with highest probability as DdrB homologs group with sequences from UniRef (Clusters #1 and #2), while the nine sequences of the DV-metagenome-only Cluster #5 have much lower probability hits to DdrB (*E*-value of 34; Supplementary 14A). All nine sequences from Cluster #5 are essentially identical, and pairwise alignment of the most complete representatives with DdrB from *D. radiodurans* finds few fully-conserved positions (Supplementary 14B).

### Dry Valley metagenomes encode a plethora of DNA ligase proteins

DNA ligases are enzymes that join breaks in the phosphodiester backbone of DNA and have an essential role in DNA replication and repair in all organisms (Williamson and Leiros, 2020). The ATP-dependent sub-class of ligases have dedicated repair functions in bacteria and are often involved in alternative stationary-phase pathways in species that have spore-forming or dormant life-phases (Pergolizzi et al., 2016; Williamson et al., 2016). Because these enzymes are known to have a range of appending domains that impart specific functions, these sequences were sub-divided by searches for additional domains.

**Figure 6:**
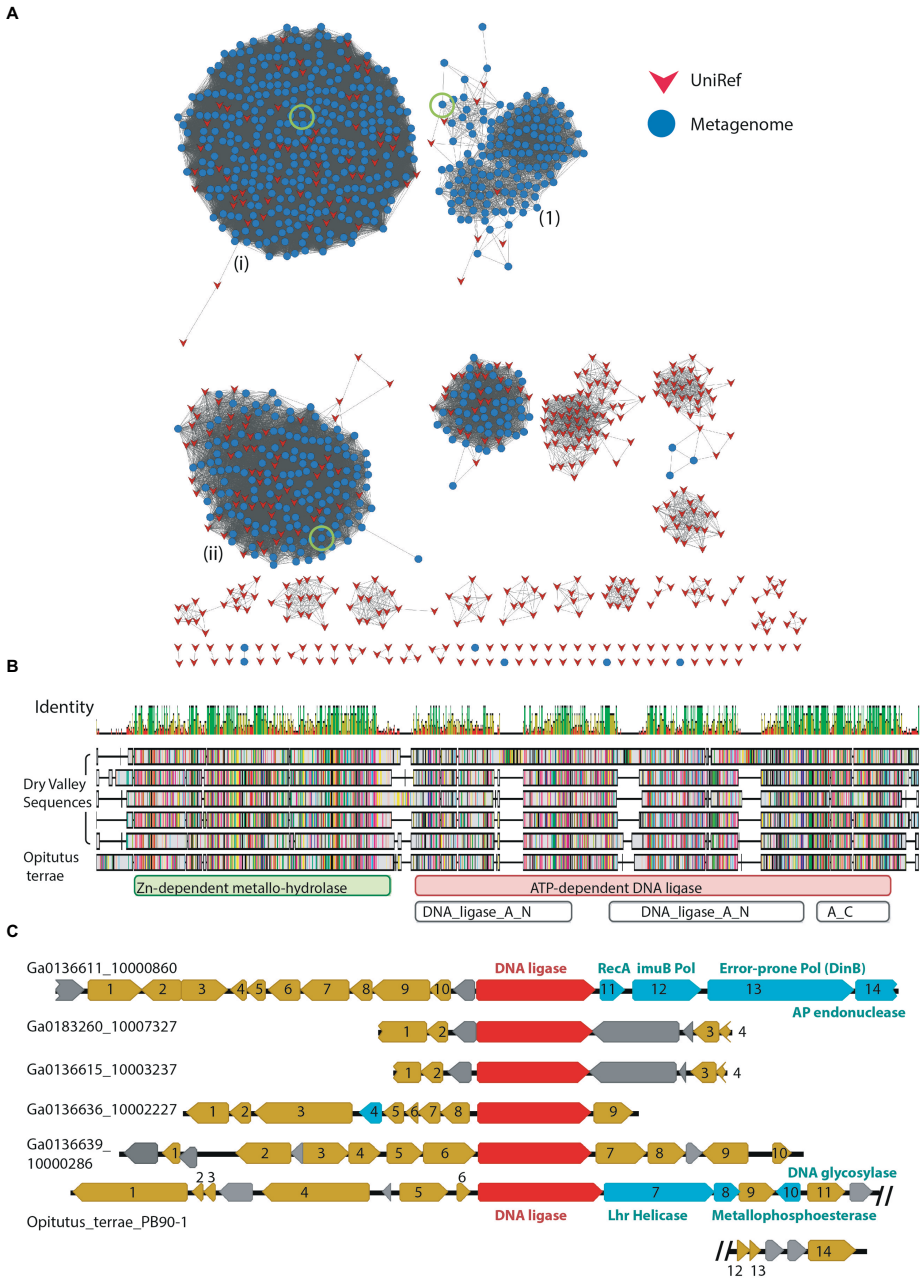
**(A)** SSN of metagenome hits to LigB-type DNA ligases at 50% identity edge threshold; other network parameters are detailed in Table 2. Domain compositions include the catalytic DNA_ligase_A_M domain together with the N-terminal DNA binding domain DNA_ligase_A_N. Dry-Valley metagenome nodes are colored blue, UniRef50 nodes are indicated in red. Cluster numbers discussed in the text are given in parentheses. The sequences used in further analysis on Clusters #1, ii and iii are indicated by a green circle. **(B)** Sequence alignment of full-length DV-metagenome sequences from Cluster #1 where an N-terminal metallonuclease (RMMBL) domain was detected with hmmscan Sequences are aligned to the *Opititus terrae* DNA ligase gene OTER_RS15935 from the genome sequence NC_010571_Opitutus_terrae_PB90-1. Domain boundaries from Pfam/Interpro sequences searches are shown below the alignment. **(C)** Genomic context of metallonuclease-ligase fusion proteins from DV-metagenomes and *O. terrae*. The nuclease-ligase gene is shown in red, putative DNA-repair genes in cyan, other annotated genes in gold and hypothetical proteins in gray.

The LigB DNA ligases are the most widespread form of bacterial ATP-dependent DNA ligase, being found in species of *Mycobacterium* and *Pseudomonas* as well as cyanobacteria such as *Prochlorococcus marinus* (Gong et al., 2004; Williamson et al., 2016; Ejaz and Shuman, 2018; Williamson and Leiros, 2019). These ligases are typically found as part of an operon which also includes an Lhr helicase, a phosphodiesterase and a metallo-beta-lactamase-fold protein with putative nuclease function (Ejaz and Shuman, 2018). The precise biological role and DNA-damage(s) targeted by this pathway are not currently known. Of the 1,791 DV-metagenome proteins which included the N-terminal DNA-binding domain of the LigB class DNA_ligase_A_N (PF04675), most grouped together with a large number of UniRef sequences at the 50% threshold (Figure 6A). Of interest, Cluster #1 has fewer UniRef representatives than other clusters, and includes DV-metagenome sequences that are significantly longer than others in the network (Supplementary 15). Additional searches with hmmscan identified 13 sequences in this cluster that contain a fusion of the Zn-dependent metallo-hydrolase RNA specificity domain RMMBL (PF07521) at the N-terminus of the DNA ligase DNA-binding domain (Figure 6B). Such fusions of the operon-associated exonuclease have been previously identified in the genome of the anaerobic bacterium *Opititus terrae* (Ejaz and Shuman, 2018), although the role of this module either as a ligase-fused domain or an autonomous enzyme has not been determined. To investigate whether these fused ligase-nuclease proteins are part of conserved operons, we compared the genomic context of a selection of these proteins where the sequence contig included flanking genes. None of the Dry Valley ligase-nuclease genes were found in the LigB/Lhr helicase/ phosphodiesterase/metallo-beta-lactamase configuration seen in the *O. terrae* genome and there was little overall synteny between clusters (Figure 6C). Several genetic regions include genes involved in protein expression such as sigma factors, transcription factors, ribosomal subunit proteins and enzymes involved in tRNA or rRNA modification (Supplementary 16) and none were predicted to be complete or partial phage. Interestingly, contig Ga0136611_10000860 two predicted error-prone DNA polymerases as well as a putative RecA-domain protein which could cooperate in DNA-repair processes.

LigD DNA ligases are involved in stationary-phase double-strand break repair as an alternative to homologous-recombination under conditions where a second partially-replicated chromosome may not be available (Pitcher et al., 2007b; Shuman and Glickman, 2007). At a bare minimum these modular enzymes have the ATP-dependent DNA ligase adenylation (DNA_ligase_A_M, PF01068) and oligonucleotide binding (DNA_ligase_A_C, PF04679) domains together with a PrimPol (PrimaseS, PF01896) domain, for example the LigD protein of *Bacillus subtilis* (Weller et al., 2002). Others additionally have a phosphodiesterase domain (LigD_N, PF13298), for example LigD proteins of *Mycobacterium tuberculosis* and *Agrobacterium tumefaciens* (Zhu and Shuman, 2007; Wright et al., 2010). Within the DV-metagenomes LigD sequences were defined as those possessing the PrimaseS and/or the LigD_N domain in addition to the conserved DNA_ligase_A_M domain. A total of 1,805 such sequences were retrieved from the DV-metagenomes, all of which were annotated as LigD by IMG (Table 2). Of these, 650 were retained after applying length cutoffs and were used to build the SSN. DV-metagenome-only clusters that formed at a 50% threshold were analyzed using hmmscan which, in addition to the PrimaseS and DNA_ligase_A_M domains, detected the oligo-nucleotide binding domain DNA_ligase_A_C that forms part of the DNA ligase catalytic core (Table 3). Clusters comprise combinations of either the DNA_ligase_A_M -DNA_ligase_A_C core domains with PrimaseS [Supplementary 17, Cluster (1)], or these core domains with LigD_N [Supplementary 17, Cluster (2)–(5)]. The absence of additional domains or sequence features, coupled with the known primary-sequence diversity inherent in the DNA-ligase family (Williamson et al., 2016) suggests these metagenome-only clusters represent partial sequences of LigD proteins, rather than unique Dry Valley variants. Full-length metagenome sequences which include both PrimaseS and LigD_N modules cluster together with UniRef sequences.

ATP-dependent DNA ligases that lacked either the Lig-B or Lig-D type domains were processed as a single dataset. The majority of sequences still clustered together with UniRef sequences at the 54% threshold (Supplementary 18). In the largest cluster [Supplementary 18. Cluster (i)] both UniRef and metagenome sequences appeared to be the LigC type DNA ligases including only the core catalytic adenylation (DNA_ligase_A_M, PF01068) and oligonucleotide binding domain (DNA_ligase_A_C, PF04679; Table 3). In other mixed clusters, UniRef ligases were the LigD or LigB-type, indicating that metagenome sequences in these clusters represented incomplete gene fragments for these enzymes. Hmmscan of the largest predominantly-metagenome clusters revealed that Clusters (1) to (3) were likewise catalytic core-only proteins (DNA_ligase_A_M and DNA_ligase_A_C) without any accessory domains, while the fourth cluster (Cluster (4)) had highest probability as a protein translocase subunit. This suggests that the majority of proteins in this dataset were LigC type proteins, some of which have considerable sequence divergence from highly-represented proteins in the UniRef database, while the remainder represent fragments of other previously-characterized DNA ligase classes.

### Dry Valley metagenomes include diverse excision-repair and direct damage-reversal proteins

The Pfam HhH-GPD comprises an expansive superfamily including various enzymes involved in DNA repair and mismatch correction such as the 8-oxoguanidine DNA glycosylases, methyl-CPG-binding proteins and methyladenine glycosylases (Bruner et al., 2000; Otani et al., 2013; Pidugu et al., 2021). The separation of distinct clusters in this network at a relatively low identity threshold (25%) likely reflects this diversity with the two largest clusters being 1,766 and 1,137 nodes and including a mixture of both metagenome and UniRef sequences [Supplementary 19 (i) and (ii)]. Separate clusters of mainly DV-metagenomic sequence composition were also identified, all of which had IMG annotations consistent with known DNA repair functions (Table 3). In an attempt to reduce the complexity of the HhH-GPD network, enzymes involved in alkylation damage repair and possessing the additional AlkA_N domain as well as the HhH-GPD domain were analyzed in a separate network (Supplementary 20). Most DV-metagenomic sequences in this network fell into the large mixed cluster together with many bacterial UniRef representatives [Supplementary 20 (1)] while a smaller cluster containing most of the remaining DV-metagenomic sequences included only two UniRef nodes [Supplementary 20 (2)]. Both clusters were annotated as DNA-3-methyladenine glycosylases by IMG (Table 3). The clustering of unique DV-metagenome sequences separate from other representatives in the UniRef database suggests that these proteins may possess unique structural feature which may be explored further.

The majority of DV-metagenome photolyases grouped with UniRef sequences clusters (i) and (ii) including 2,303 and 430 DV-metagenome sequences, respectively, (Supplementary 21). Both clusters include a combination of eukaryotic and prokaryotic sequences which are predicted to be photolyases, and most DV-metagenome sequences in these clusters are annotated in IMG as photolyases. Hmmscan analysis of the three DV-metagenome-only clusters Cluster #1-#3 indicates that these have non-DNA repair functions as copper ion transporters, haloacid dehalogenases and regulatory NAD-dependent protein deacetylases (Table 3). The large number of photolyase genes annotated in the DV-metagenome sequences indicates that the direct reversal mechanism of removing UV-induced pyrimidine-pyrimidine lesions is important in these organisms which is consistent with the findings of previous studies which have implicated the high activity of photolyase proteins in extreme UV resistance of bacteria isolated from low-temperature environments (Albarracin et al., 2014; Marizcurrena et al., 2017).

### Recombinant expression and preliminary characterization of novel nuclease and ligase genes

To gain further insight into potential DNA repair activities of proteins identified by SSN analysis, we attempted recombinant expression of metagenomic proteins with interesting sequence features. Proteins chosen for further investigation, together with the conditions for their production are summarized in Table 4. Briefly, heterologous protein expression using *E. coli* BL21pLysS was achieved for a putative Cluster (2) NucS (hereafter DV-NucS), an Hjc/UPF0102 homolg (hereafter DV-Hjc) and two LigB DNA ligases (hereafter DV-Lig-2 and DV-Lig-5). DV-metagenome proteins were expressed as fusions with the MBP solubilization tag which was successfully removed by digestion with TEV protease in all cases, with the exception of DV-Lig-2 (Supplementary 22, 23). Production of the full-length nuclease-LigB ligase protein was unsuccessful due to low expression levels and poor solubility (data not shown), therefore we attempted expression of the two predicted enzymatic modules, the RMMBL nuclease (hereafter DV-1-1-Nuc) and the LigB ATP-dependent DNA ligase (hereafter DV-1-1-Lig) separately (Supplementary 24A). The new construct for DV-1-1-Lig with the N-terminus at amino acid Asp 400 of the nuclease-ligase interdomain linker had high soluble expression in Origami (DE3) cells and was readily purified *via* a non-cleavable N-terminal His-tag (Supplementary 24C). In contrast, the nuclease domain, while readily expressed, was insoluble as both His-tagged and MBP-tagged constructs (Supplementary 24B). Attempts at soluble production of the UvdE and Rad52_Rad22 homologs from the DV-metagenome were unsuccessful, resulting in inclusion bodies (data not shown).

**Table 4:** Summary of proteins recombinantly expressed from DV-metagenome.

Name	Gene ID	Domains	Predicted molecular weight (kDa)	Expression strategy	Enzyme activity
DV-NucS	Ga0136640_100017415	NucS	58.8	MBP fusion, TEV-cleaved	Endonuclease (uracil and abasic), exonuclease single-stranded
DV-Hjc	Ga0136632_1000003432	UPF0102/Hjc	14.0	MBP fusion, TEV-cleaved	Not detected
DV-Lig-2	Ga0136636_1000055115	DNA_ligase_A_N, DNA_ligase_A_M, DNA_ligase_A_C	59.3	MBP fusion	DNA ligase
DV-Lig-5	Ga0136613_1000000468	DNA_ligase_A_N, DNA_ligase_A_M, DNA_ligase_A_C	55.0	MBP fusion, TEV-cleaved	DNA ligase
DV-1-1-Nuc-Lig	Ga0136611_1000086013	RMMBL, DNA_ligase_A_N, DNA_ligase_A_M, DNA_ligase_A_C	105.6		Not tested
DV-1-1-Nuc	Ga0136611_1000086013 (nt 1–1,197; aa 1–399)	RMMBL	44.0	Insoluble	Not tested
DV-1-1-Lig	Ga0136611_1000086013 (nt 1,198–2,874; aa 400–959)	DNA_ligase_A_N, DNA_ligase_A_M, DNA_ligase_A_C	62.0	N-terminal His-tag	DNA ligase
DV-UvdE	Ga0136640_100700761	UvdE	34.1	Insoluble	Not tested
DV-DdrA	Ga0136611_1000290011	Rad52_Rad22	44.9	Insoluble	Not tested

**Figure 7:**
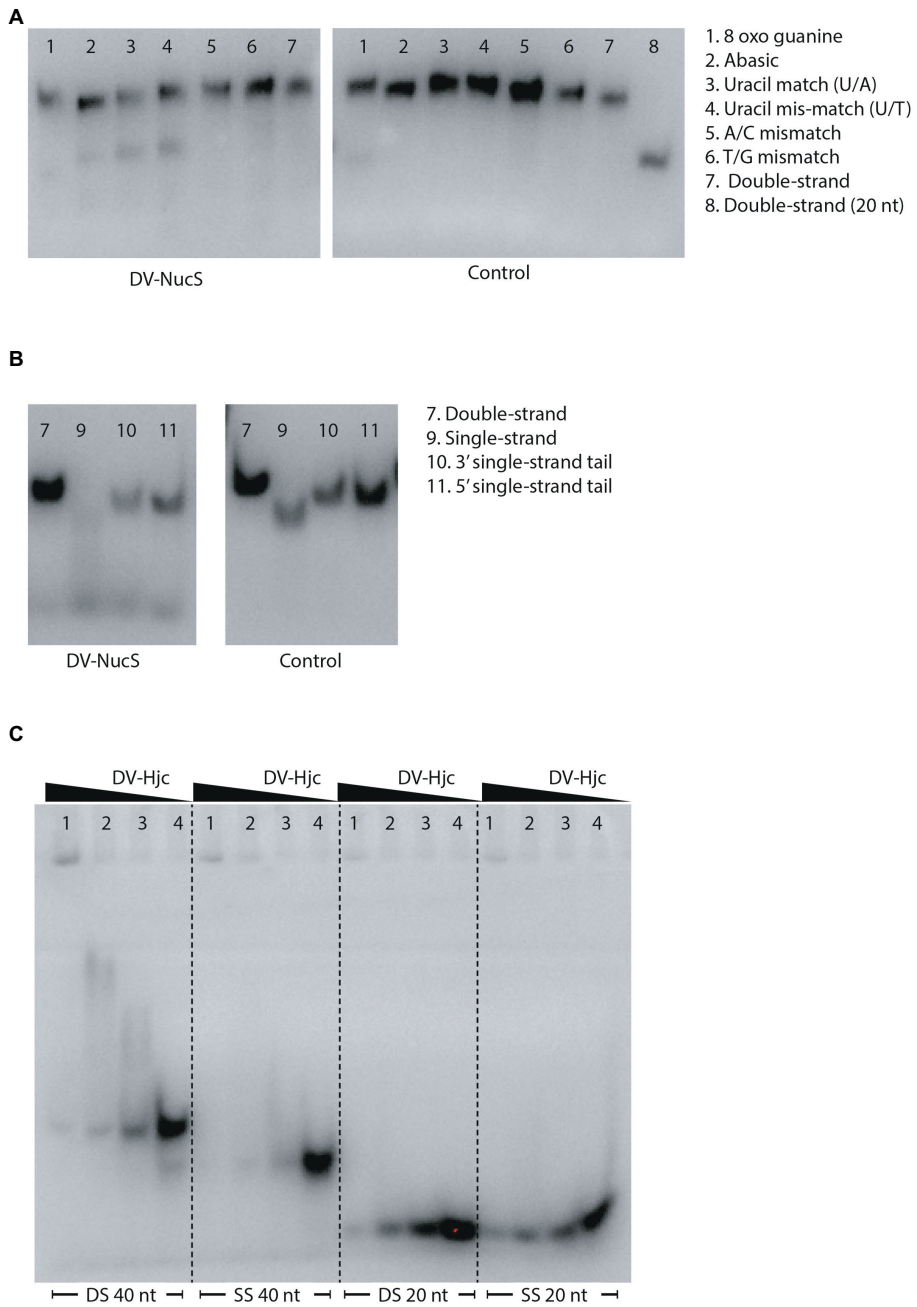
**(A)** Nucleolytic activity of DV-NucS on double-stranded DNA substrates containing damaged or mis-matched bases at a central position as shown in Supplementary 4. Substrates were incubated for 18 h with 344 nM DV-NucS (DV-NucS gel) or an equivalent volume of buffer (control gel) at 25°C prior to analysis by denaturing urea PAGE. **(B)** Nucleolytic activity of DV-NucS on DNA substrates with double and single-stranded sections as shown in Supplementary 5. Substrates were incubated for 18 h with 344 nM DV-NucS (DV-NucS gel) or an equivalent volume of buffer (control gel) at 25°C prior to analysis on 10% native TBE gels. **(C)** DNA binding activity of DV-Hjc to 40 bp double-stranded DNA (DS 40 nt), 40 nucleotide single-stranded DNA (SS 40 nt), 20 bp DNA (DS 20 nt) and 20 nucleotide single-stranded DNA (SS 20 nt). DV-Hjc concentrations were 3.3 mM (lane 1), 657 nM (lane 2) and 329 nM (lane 3) with and storage buffer as a control (lane 4). DV-Hjc dilutions were pre-incubated with substrates at 15°C for 30 min prior to electrophoresis on 10% native TBE gels with a 7% stacking layer.

Nucleolytic activity of DV-NucS and DV-Hjc was tested against a range of double-stranded DNA substrates incorporating various DNA damages and mismatches as well as 5′/3′ tailed single-stranded/ double-stranded duplexes and junctions (listed in tables Supplementary 6, 7, and shown schematically in Supplementary 4, 5). Nuclease activity was detected for DV-NucS with single-stranded DNA as well as site-specific cutting of duplexes containing an abasic site, matched uracil (opposite adenine) and mismatched uracil (opposite thymine) (Figures 7A,B). The previously-described mismatch cleaving activity of *Corynebacterium glutamicum* NucS/EndoMS required the presence of the beta-sliding clamp (DnaN subunit), and it is possible that similar additional factors may promote mismatch cleavage by DV-NucS (Ishino et al., 2018; Wozniak and Simmons, 2022). It is likewise possible that the uracil and abasic site cleavage activity of DV-NucS, which was observed after extended incubations, would be further increased by the inclusion of binding partners. Specific cleavage of uracil and hypoxanthine-containing sites, which are the product of deamination of cytosine and adenine, respectively, were recently described in NucS from the radiation-resistant archaeon *Thermococcus gammatolerans* (Zhang et al. 2020a,b) and DV-NucS may function. as part of a similar deamination-removal pathways. The apparent single-stranded degrading activity of DV-NucS is surprising and this observation should be further investigated subsequent to confirming this activity *via* construction of active-site null mutants.

DV-Hjc exhibited no nucleolytic activity on any of the substrates under the conditions tested (Supplementary 25A–C). Thermal denaturation measured by DSF shows that purified DV-Hjc unfolds with a two-state transition with a *T*_m_ between 41 to 50°C depending on pH, indicating that this protein possesses a well-folded hydrophobic core and is relatively stable under assay conditions making mis-folding during the production process an unlikely explanation for the lack of activity (Supplementary 25D). Likewise, EMSA indicates DV-Hjc binds strongly to both single- and double-stranded DNA, suggesting it does function in some DNA-modifying process (Figure 7C). It is possible that DV-Hjc requires assay conditions other than those tested here, for example different metals, cofactors or interaction partners. It is also possible that, despite its homology to resolvases, DV-Hjc does not function as a nuclease but plays some other function in its native host such as DNA protection, recruitment of other nucleic-acid modifying enzymes to DNA or transcription factor activity.

To investigate whether DV-NucS or DV-Hjc are part of operons or gene clusters that include potential interaction partners, we analyzed the synteny of four of the largest contigs to detect common adjacent genes. A putative lysophospholipase was directly upstream of all four DV-NucS genes, however there were no other notably conserved adjacent open reading frames (Supplementary 26A). All five DV-Hjc genes were flanked by a predicted RNAseHII gene but again there was relatively low conservation. In other surrounding genes (Supplementary 26B).

**Figure 8:**
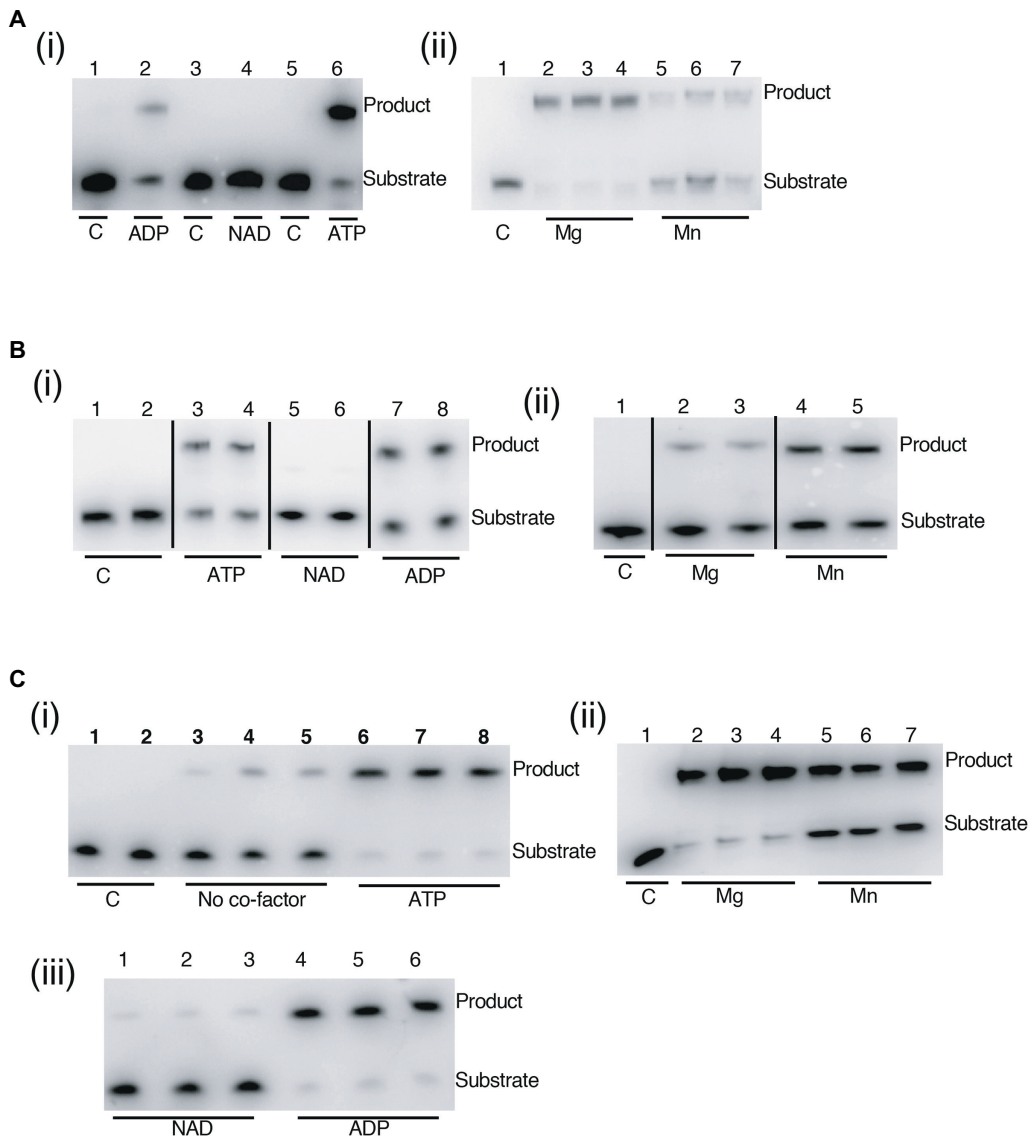
DNA ligase activity of LigB-type enzymes on singly nicked substrates. **(A)** DV-Lig-2 activity. Numbers represent single samples in each lane in panel (i), while lanes 2–4 and 5–7 in panel (ii) are triplicate reactions with of Mg and Mn, respectively. **(B)** DV-Lig-5 activity. Lanes 3–4, 5–6 and 7–8 in panel (i) are duplicate reactions with ATP, NAD and ADP, respectively, and lanes 2–3 and 4–5 in panel (ii) are duplicates with Mg and Mn. **(C)** DV-1-1-Lig activity. Lanes 1–2, 3–5, and 6–8 in panel (i) are duplicates of the control or triplicates of the no-cofactor and ATP-controls, respectively. Lanes 2–4 and 5-7in panel (ii) are triplicates with Mg. and Mn. Lanes 1–3 and 4–6 are triplicates with NAD and ADP respectively. Final enzyme concentrations were 100 nM (DV-Lig-5 and DV-1-1-Lig) and 100 nM (DV-Lig-2, estimated as a 50:50 ratio cleaved:uncleaved from the SDS-PAGE gel). Concentrations of Mg and Mn were 10 mM and nucleotide concentrations were 1 μM of ATP, ADP or NAD+ as indicated. Nucleotide cofactor preference assays [panels (i)] were run with Mg; divalent cation preference assays [panels (ii)] were all run with ATP. All reactions were incubated at 20°C for 2 h prior to analysis by denaturing Urea-PAGE gel electrophoresis; the nucleotide cofactor reaction was pre-incubated with unlabeled DNA as described in the methods. C indicates no-protein control in all figures.

Assay of DV-Lig-2, DV-Lig-5 and the DV-1-1-Lig domain with nicked DNA showed that all three enzymes function as DNA ligases in the presence of ATP (Figure 8). Both DV-Lig-5 and DV-1-1-Lig also exhibit considerable activity with ADP as an adenylate donor while DV-Lig-2 has some ligation activity with ADP, although it is considerably lower than that observed with ATP. Similar dual usage of these cofactors has been observed for many ATP-dependent DNA ligases, typically with a preference for the nucleotide triphosphate form over the diphosphate (Jeon and Ishikawa, 2003; Williamson and Pedersen, 2014). Neither DV-Lig-2 nor DV-Lig-5 ligate DNA in the presence of NAD^+^, while the small amount of ligation observed from DV-1-1-Lig. This activity with NAD was similar to the no-cofactor control background, indicating that some purified enzyme-adenylate remained despite the pre/incubation step with unlabeled DNA rather than indicating the use of NAD by this enzyme. A comparison of magnesium and manganese show both are used as cofactors by all three enzymes, however DV-Lig-2 and DV-1-1-Lig have considerably higher activity with magnesium while DV-Lig-5 has a preference for manganese.

## Conclusion

In summary, our sequence homology approach using SSN analysis identified a large number of known genes known to be associated with repair functions, for example from *D. radiodurans* and mycobacteria. In addition, we have identified a several new genes which appear to be unique to this environment, or have very few representatives in current databases. Activity assays on recombinantly-produced enzymes encoded by these genes have demonstrated their DNA-processing abilities, validating this approach as a method to identify novel DNA repair or replication enzymes in metagenomic datasets. As the volume of DNA sequence data continues to increase, so too do the opportunities to discover new and fascinating aspects of the microbial diversity that inhabit our planet, making approaches necessary that can evaluate the true functional extent of this diversity (Temperton and Giovannoni, 2012). The integrated application of biochemical and *in silico* approaches to unique samples, such as those in this study, has significant potential impacts ranging from experimental validation of hypothetical protein functions, through to the description of entirely new DNA repair mechanisms underpinning survival in these highly adapted communities. The present work serves to enhance our general understanding of microbial adaptation to DNA-damaging environments. Further, as many molecular biological protocols utilize DNA modifying enzymes, there is enormous potential for the development of biotechnological tools from newly discovered activities, which may be facilitated by this approach.

## Data availability statement

The datasets presented in this study can be found in online repositories. The names of the repository/repositories and accession number(s) can be found in the article/Supplementary material.

## Author contributions

AW and SC conceived, designed, and coordinated the study. MM and SC selected sample sites for sequencing, purified, and sequenced the metagenomic DNA. ER-S, RS, and AW recombinantly-produced and assayed enzymes. ER-S and AW drafted the manuscript. All authors contributed to the article and approved the submitted version.

## Funding

This work was supported by the The Marsden Fund of New Zealand (18-UOW-034) to AW, ER-S, and MM are supported by University of Waikato Doctoral Scholarships, RS was supported by a University of Waikato Summer Studentship. ER-S received a University of Waikato School of Science Student Trust Research Grant toward consumables. The metagenomic sequencing of the Dry Valley samples was supported through awards to SC from The Community Sequencing Program (The Joint Genome Institute, DOE, USA) and The New Zealand Antarctic Research Institute (NZARI). Logistics support for the field seasons was provided by Antarctica New Zealand.

## Conflict of interest

The authors declare that the research was conducted in the absence of any commercial or financial relationships that could be construed as a potential conflict of interest.

## Publisher’s note

All claims expressed in this article are solely those of the authors and do not necessarily represent those of their affiliated organizations, or those of the publisher, the editors and the reviewers. Any product that may be evaluated in this article, or claim that may be made by its manufacturer, is not guaranteed or endorsed by the publisher.

## Footnotes

^1^ https://jgi.doe.gov/user-programs/pmo-overview/project-materials-submission-overview/

^2^ https://img.jgi.doe.gov/cgi-bin/mer/main.cgi

^3^ http://hmmer.org/

^4^ https://efi.igb.illinois.edu/efi-est/

^5^ https://cytoscape.org/release_notes_3_8_2.html
