# Supplementary material for: DNA repair enzymes of the Antarctic Dry Valley metagenome
Source: Front Microbiol. 2023 Apr 14;14:1156817. doi: 10.3389/fmicb.2023.1156817 (PMC10140301; doi:10.3389/fmicb.2023.1156817)

Supplementary Material

DNA Repair Enzymes of the Antarctic Dry-Valley Metagenome

Elizabeth Rzoska-Smith, Ronja Stelzer, Maria Monterio, Craig S Carey and Adele Williamson*

*** Correspondence:**
Adele Williamson
adele.williamson@waikato.ac.nz

1 Supplementary Figures and Tables

Supplementary 1. GOLD Analysis IDs and total gene counts of metagenomes in JGI database for each sample. Gene counts for sequences discussed in detail are given at each site. NucS counts are for Cluster 2; Hjc are for Clusters 1 and 2; UvsE are for clusters 1 and 2; Rad52_22 are for Clusters 1 and 2; DdrB are for clusters 1, 2 and 5; LigB is for Cluster 1.

Genome Name	GOLD Analysis Project ID	Gene Count (assembled)	Total count						Normalized to gene count (per $1x10^6$)					
			NucS	Hjc	UvdE	Rad52_22	DdrB	LigB	NucS	Hjc	UvdE	Rad52_22	DdrB	LigB
UQ134	Ga0136610	1345267			43						32.0			
UQ223	Ga0136611	3300976	3	41		36		8	0.9	12.4		10.9		2.4
UQ224A	Ga0136612	2600309		19	9	12		7		7.3	3.5	4.6		2.7
UQ272	Ga0136613	2801457	8	41	21	25		10	2.9	14.6	7.5	8.9		3.6
UQ279	Ga0136614	4333264			35						8.1			
UQ288	Ga0136615	1996511	7	30	14	32		4	3.5	15.0	7.0	16.0		2.0
UQ299	Ga0136616	2120495	6	29	15	32		4	2.8	13.7	7.1	15.1		1.9
UQ322	Ga0136617	5011667	10	59	35	64		22	2.0	11.8	7.0	12.8		4.4
UQ330	Ga0136618	1850995	5	29	9	21		4	2.7	15.7	4.9	11.3		2.2
UQ353	Ga0136619	1523026	6	21	7	18		4	3.9	13.8	4.6	11.8		2.6
UQ416	Ga0136620	1721004	4	30	10	22		8	2.3	17.4	5.8	12.8		4.6
UQ445A	Ga0136621	1448922	4	19	4	10		5	2.8	13.1	2.8	6.9		3.5
UQ448	Ga0136622	1749225	1	34	9	19		7	0.6	19.4	5.1	10.9		4.0
UQ449	Ga0136623	1696950	1	19	6	13	9	9	0.6	11.2	3.5	7.7	5.3	5.3
UQ469	Ga0136624	1163852		24	6	10	9	1		20.6	5.2	8.6	7.7	0.9
UQ483	Ga0136625	1299321			2	9		1			1.5	6.9		0.8
UQ487	Ga0136626	46280												
UQ489	Ga0136627	1108965	5	20	5	14		1	4.5	18.0	4.5	12.6		0.9

UQ497	Ga0183260	3620202	3	50	20	28		8	0.8	13.8	5.5	7.7		2.2
UQ540	Ga0180121	1552546	4	20	5	14		3	2.6	12.9	3.2	9.0		1.9
UQ568	Ga0136630	1385786	1	31	7	15		5	0.7	22.4	5.1	10.8		3.6
UQ601	Ga0136631	1667134	1		3	11		2	0.6		1.8	6.6		1.2
UQ611	Ga0136632	1839415	2	26	9	15		11	1.1	14.1	4.9	8.2		6.0
UQ83	Ga0136633	1296057		17	6	8		8		13.1	4.6	6.2		6.2
UQ833	Ga0136634	1800040	6	44	7	17	8		3.3	24.4	3.9	9.4	4.4	
UQ85	Ga0136635	1394326	2	15	5	9			1.4	10.8	3.6	6.5		
UQ852	Ga0136636	1601818	1	45		3			0.6	28.1		1.9		
UQ857	Ga0136637	1279577	6	26	8	14		2	4.7	20.3	6.3	10.9		1.6
UQ858	Ga0136638	2036446	6	34	13	28		5	2.9	16.7	6.4	13.7		2.5
UQ859	Ga0136639	1463554	17	27				2	11.6	18.4				1.4
UQ864	Ga0136640	1634979	3	43	11	31		4	1.8	26.3	6.7	19.0		2.4

Supplementary 2. Primers used to generate truncated forms of DV-1-1-Nuc-Lig with only the nuclease or ligase domains separately

Primer name:	Primer sequence*
DV1-1Nuc Forward	**<u>GAGAACCTGTATTTTCAGGGTCATCGT</u>**
DV1-1Nuc Back	GGGGACCACTTTGTACAAGAAAGCTGGGTATAA**<u>ACCCGGTGCGCTAGG</u>**
DV1-1Lig Forward	GAGAACCTGTATTTTCAGGGT**<u>GATTTTGCACGTTTTGCC</u>**
DV1-1Lig Back	**<u>GGGGACCACTTTGTACAAGAAAGCTGGGTCTTATTCGGTATCTGCTTT</u>**
DV1-1 Forward- 2	GGGGACAAGTTTGTACAAAAAAGCAGGCTTA**<u>GAGAACCTGTATTTTCAGGGT</u>**

* Binding region indicated by bold underlined text

Supplementary 3. Optimized expression conditions used for protein production in this study. No conditions promoting soluble expression were identified for DV-DdrA, DV-UvdE or DV-1-1-Nuc-Lig or DV-1-1-Nuc

Protein	Expression conditions			Purification conditions	
	Vector	*E. coli* strain	Temperature	TEV cleavage	Gel filtration column
DV-NucS	pHMGWA	BL21 (DE3) pLysS	15 °C	Yes	S200
DV-Hjc	pHMGWA	BL21 (DE3) pLysS	20 °C	Yes	S75
DV-Lig-2	pHMGWA	Origami (DE3)	15 °C	No	S200
DV-Lig-5	pHMGWA	Origami (DE3)	15 °C	Yes	S200
DV-1-1-Lig	pDEST17	Origami (DE3)	15 °C	No	S200

Supplementary 4. Schematic of enzyme assays for nuclease activity on damage/mismatch DNA substrates or DNA ligase activity using fluorescently-labelled oligonucleotide substrates. Stars represent labelling with the 6-carboxyfluorescein at the 5' terminus (5'FAM). Labelled strands are indicated by a black line while unlabeled portions of substrate duplexes are not visible during analysis are indicated by grey lines. For a list of oligonucleotides used to generate these substrates see **Supplementary 6** and **Supplementary 7.** A) Analysis of assay products by denaturing TBE-urea PAGE indicating separation of oligonucleotide strands and electrophoretic detection of the labelled strands on the gel (yellow boxes). B) Design of double-stranded substrates incorporating damaged bases or mismatches at a central position and the predicted outcomes of endonuclease activity. C) Design of substrates incorporating single-stranded flaps, splays, or double-stranded junctions. D) Design f DNA ligase substrate with single nick.

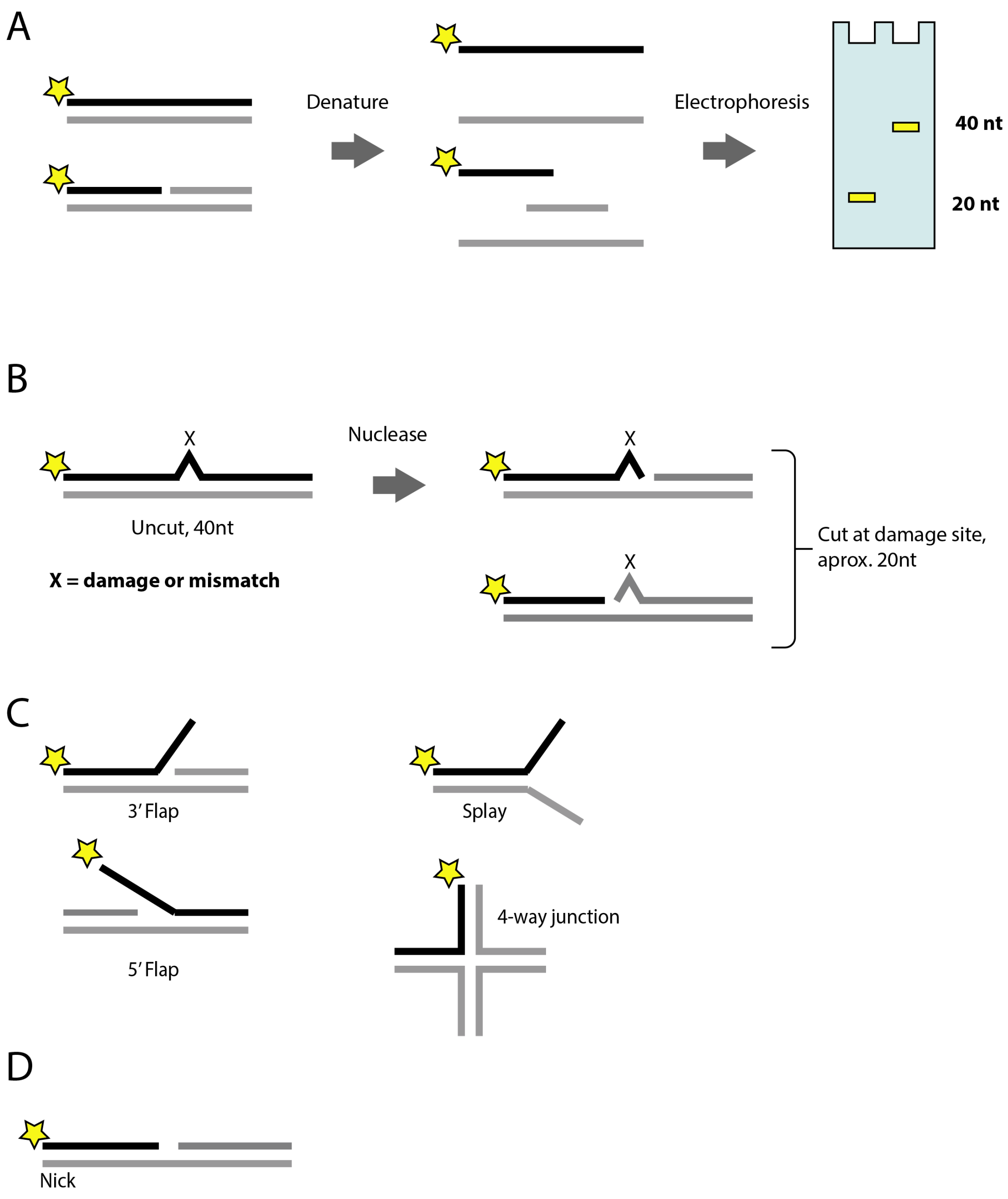

Supplementary 5. Schematic of enzyme assays for nuclease activity on DNA substrates with carrying single-stranded portions. Stars represent labelling with the 6-carboxyfluorescein at the 5' terminus (5'FAM). Labelled strands are indicated by a black line while unlabeled portions of substrate duplexes are not visible during analysis are indicated by grey lines. For a list of oligonucleotides used to generate these substrates see Supplementary 6 and Supplementary 7. A) Analysis of assay products by native TBE PAGE indicating a size-shift based on degradation of any part of the duplex (yellow boxes). B) Design of substrates with double and single-stranded portions.

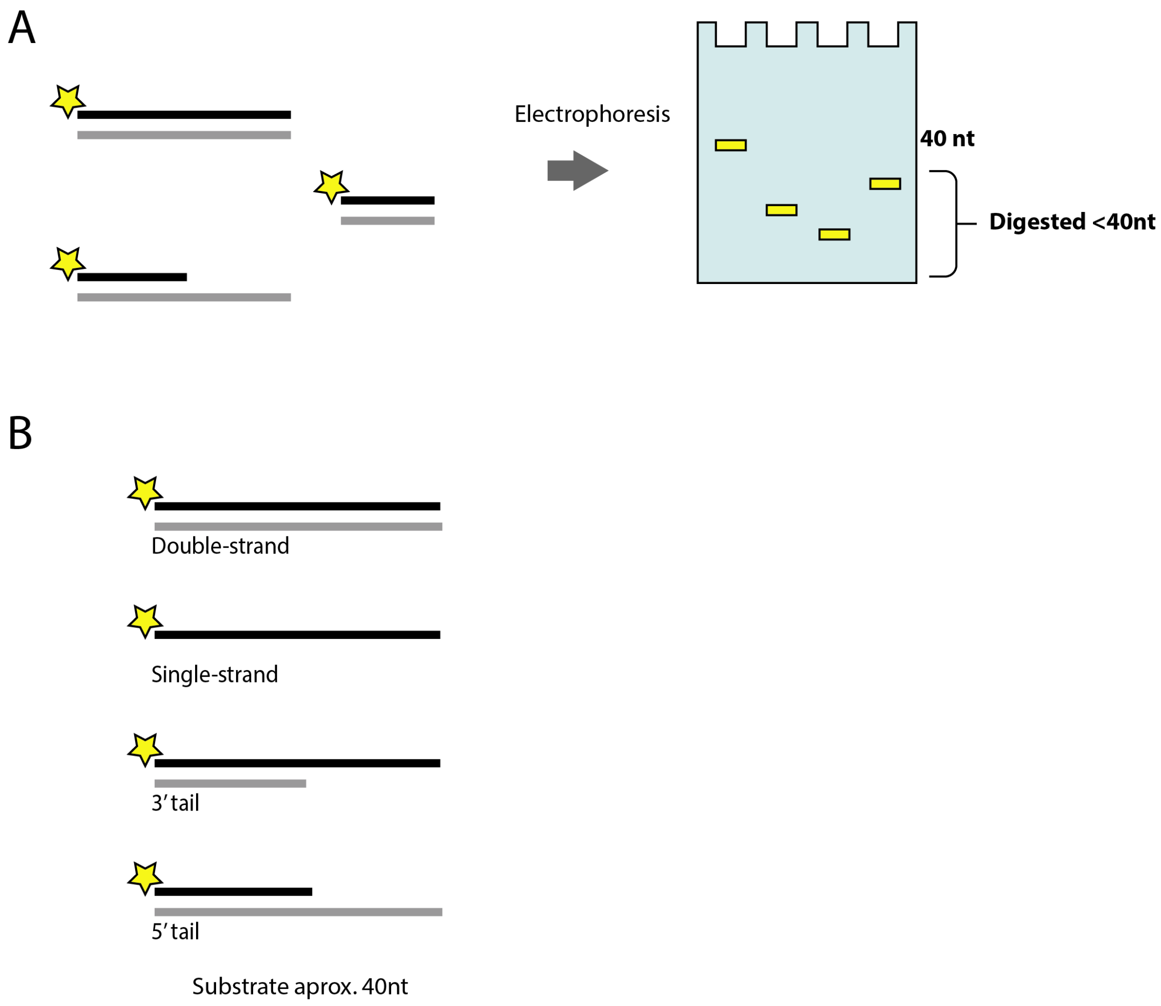

Supplementary 6. DNA oligomer sequences used to construct assay substrates given in Supplementary 7. Abbreviations:5' 6-carboxyfluorescein (5'FAM) 8-Oxo-deoxyguanosine (8-Oxo-dG), abasic tetrohydrofuran (dSpacer).

Name	Sequence (5' to 3')	Modifications
NL1	AGGCCATGGCTGATATCGCA	5' FAM
NL3	CGACGGAGCTCGAATGCCTATGCGATATCAGCCATGGCCT	
NL5	AGGCCATGGCTGATATCGCATAGGCATTCGAGCTCCGTCG	5' FAM
NL6	CGACGGAGCTCGAATGCCTA	
NL10	CGACGGAGCTCGAATGCCTACGCGATATCAGCCATGGCCT	
MD5	AGGCCATGGCTGATATC**X**CATAGGCATTCGAGCTCCGTCG	5' FAM, X=8-Oxo-dG
MD6	AGGCCATGGCTGATATCGCA**X**AGGCATTCGAGCTCCGTCG	5' FAM, X=dSpacer
MD9	AGGCCATGGCTGATATCGCA**U**AGGCATTCGAGCTCCGTCG	5' FAM
MD10	CGACGGAGCTCGAATGCCTGTGCGATATCAGCCATGGCCT	
HJ1	GACGCTGCCGAATTCTACCAGTGCCTTGCTAGGACATCTTTGCCCACCTGCAGGTTCACCC	5' FAM
HJ2	TGGGTGAACCTGCAGGTGGGCAAAGATGTCCTAGCAATGTAATCGTCAAGCTTTATGCCGTT	
HJ3	CAACGGCATAAAGCTTGACGATTACATTGCTAGGACATGCTGTCTAGAGGATCCGACTATCGA	
HJ4	ATCGATAGTCGGATCCTCTAGACAGCATGTCCTAGCAAGGCACTGGTAGAATTCGGCAGCGT	
HJ5	ATCATAGCTAACATGACTAGTGCGATATCAGCCATGGCCT	
HJ6	CTAGTCATGTTAGCTATGAT	

Supplementary 7. DNA oligomer combinations used to construct various assay substrates shown in Supplementary 4 and Supplementary 5. Oligonucleotide sequences are given in Supplementary 6.

**Assay**	**Substrate name**	**Oligonucleotide combinations**
DNA damage	8-Oxo guanine	MD5*, NL3[a]
	Abasic	MD6*, NL3[a]
	Uracil match	MD9*, NL3[a]
	Uracil mis-match	MD9*, MD10[a]
	A/C mismatch	NL5*, NL10[a]
	T/G mismatch	NL5*, MD10[a]
Flap/splayed	Flap 3’	NL15*, HJ6[a], HJ5[a],
	Flap 5’	NL15*, NL7[a], HJ5[a]
	Splayed	NL15*, HJ6[a]
	Holliday junction	HJ1*, HJ2[a], HJ3[a], HJ4[a]
Ligase	Nick	NL1*, NL2[b], NL3[b]
Nuclease	Single-strand	NL5*
	3’ tail	NL5*, NL6[a]
	5’ tail	NL1*, NL3[a]
Controls	Double-strand	NL5*, NL3[a]
	Double-strand (20+20)	NL1*, NL6[a]

* Labeled strand, 80 nM final concentration in assay

[a] 112 nM final concentration in assay

[b] 400 nM final concentration in assay

Supplementary 8. Counts of DNA replication and repair genes from metagenomes sequenced in this study (*) and other publically-available metagenomes/ genomes from IMG (**). Sample descriptions are taken from metadata provided to IMG.

Genome code in figures	Description	Location	Replication and repair gene count	Replication and repair % (of genes in COGs)	GOLD analysis ID
Antarctic Dry Valley metagenome*					
DV-1	UQ134	Antarctic Dry Valley	43041	5.57	Ga0136610
DV-2	UQ223	Antarctic Dry Valley	102080	5.44	Ga0136611
DV-3	UQ224A	Antarctic Dry Valley	82984	5.54	Ga0136612
DV-4	UQ272	Antarctic Dry Valley	86497	5.41	Ga0136613
DV-5	UQ279	Antarctic Dry Valley	130142	5.38	Ga0136614
DV-6	UQ288	Antarctic Dry Valley	62264	5.57	Ga0136615
DV-7	UQ299	Antarctic Dry Valley	66185	5.47	Ga0136616
DV-8	UQ322	Antarctic Dry Valley	148201	5.11	Ga0136617
DV-9	UQ330	Antarctic Dry Valley	59811	5.44	Ga0136618
DV-10	UQ353	Antarctic Dry Valley	49745	5.55	Ga0136619
DV-11	UQ416	Antarctic Dry Valley	57617	5.49	Ga0136620
DV-12	UQ445A	Antarctic Dry Valley	47184	5.51	Ga0136621
DV-13	UQ448	Antarctic Dry Valley	57680	5.49	Ga0136622
DV-14	UQ449	Antarctic Dry Valley	54763	5.44	Ga0136623
DV-15	UQ469	Antarctic Dry Valley	38508	5.83	Ga0136624
DV-16	UQ483	Antarctic Dry Valley	43257	5.76	Ga0136625
DV-17	UQ487	Antarctic Dry Valley	1608	6.05	Ga0136626
DV-18	UQ489	Antarctic Dry Valley	36089	5.79	Ga0136627

DV-19	UQ497	Antarctic Dry Valley	116217	5.59	Ga0183260
DV-20	UQ540	Antarctic Dry Valley	47977	5.01	Ga0180121
DV-21	UQ568	Antarctic Dry Valley	46370	5.66	Ga0136630
DV-22	UQ601	Antarctic Dry Valley	54991	5.48	Ga0136631
DV-23	UQ611	Antarctic Dry Valley	58045	5.33	Ga0136632
DV-24	UQ83	Antarctic Dry Valley	45650	5.52	Ga0136633
DV-25	UQ833	Antarctic Dry Valley	49755	4.96	Ga0136634
DV-26	UQ85	Antarctic Dry Valley	42656	4.97	Ga0136635
DV-27	UQ852	Antarctic Dry Valley	45086	4.90	Ga0136636
DV-28	UQ857	Antarctic Dry Valley	42459	5.79	Ga0136637
DV-29	UQ858	Antarctic Dry Valley	62787	5.21	Ga0136638
DV-30	UQ859	Antarctic Dry Valley	47859	5.33	Ga0136639
DV-31	UQ864	Antarctic Dry Valley	53989	5.50	Ga0136640
Forest soil metagenome**					
MG-1	Enriched temperate forest soil	Barre Woods Harvard Forest LTER site, Massachusetts, United States	257	5.03	Ga0206996
MG-2	Temperate forest soil	Eldorado National Forest, California, United States	212332	3.64	Ga0372949
MG-3	Temperate forest soil	Harvard Forest LTER site, Massachusetts, United States	87395	3.16	Ga0372957
MG-4	Temperate forest soil	Shasta-Trinity National Forest, California, United States	6975	3.81	Ga0233357
MG-5	Enriched tropical forest soil	Luquillo Experimental Forest, Puerto Rico	118761	3.84	Ga0318519
MG-6	Tropical forest soil	Luquillo Experimental Forest, Puerto Rico	59055	3.79	Ga0318495
Other soil/ sediment metagenome**					

MG-7	Hill slope soil	Landscape Evolution Observatory, Arizona, United States	22515	4.92	Ga0247719
MG-8	Grasslands soil	Angelo Coastal Reserve, California, United States	4659	2.51	Ga0134036
MG-9	Grasslands soil	Angelo Coastal Reserve, California, United States	48752	4.06	Ga0134111
MG-10	Mangrove sediment	Sao Paulo State, Brazil	477	1.89	Ga0066231
MG-11	Soil and rhizosphere microbial communities	Laval, Canada	2317	4.25	Ga0208760
MG-12	Enriched backyard soil	Emeryville, California, United States	33916	3.75	Ga0182747
Freshwater sediment metagenome**					
MG-13	River sediment	Broadkill River, Delaware, United States	10069	4.72	Ga0194010
MG-14	Freshwater lake sediment	Prairie Pothole Lake, North Dakota,United States	55044	4.49	Ga0105096
MG-15	Groundwater aquifer sediment	East River, Colorado, United States	43508	4.10	Ga0210378
MG-16	Freshwater lake sediment	Prairie Pothole Lake, North Dakota, United States	71943	3.99	Ga0105100
Human pathogen isolate**					
PG-1	Salmonella enterica enterica sv. Typhimurium 33676	Isolate	141	3.53	Ga0111327
PG-2	Escherichia coli PSUO2	Isolate	163	3.46	Ga0226370
PG-3	Escherichia coli M6	Isolate	160	3.97	Ga0175133
PG-4	Salmonella enterica enterica sv. Infantis CVM44454 2014am-3028	Isolate	147	3.68	Ga0175242
PG-5	Staphylococcus aureus FORC_027	Isolate	107	4.94	Ga0175076
PG-6	Mycobacterium bovis BCG Tokyo 172	Isolate	94	3.30	Ga0029473

PG-7	Mycobacterium avium 2285 (R)	Isolate	104	2.70	Ga0069436
PG-8	Salmonella enterica enterica sv. Enteritidis SEJ	Isolate	128	3.32	Ga0059155
PG-9	Escherichia coli O55:H7 USDA 5905	Isolate	143	3.58	Ga0013912
PG-10	Escherichia coli PSUO2	Isolate	163	3.46	Ga0226370
PG-11	Salmonella enterica enterica sv. Enteritidis CMCC50041	Isolate	127	3.30	Ga0125088
PG-12	Salmonella enterica enterica sv. Typhimurium 33676	Isolate	141	3.53	Ga0111327
PG-13	Escherichia coli sv. O157:H7 472	Isolate	164	3.88	Ga0175131

* Data from this study

**Data from other studies available in IMG

Supplementary 9. Counts of genes in each COG category. GOLD identifiers and descriotion of each sample is given in in **Supplementary 1**

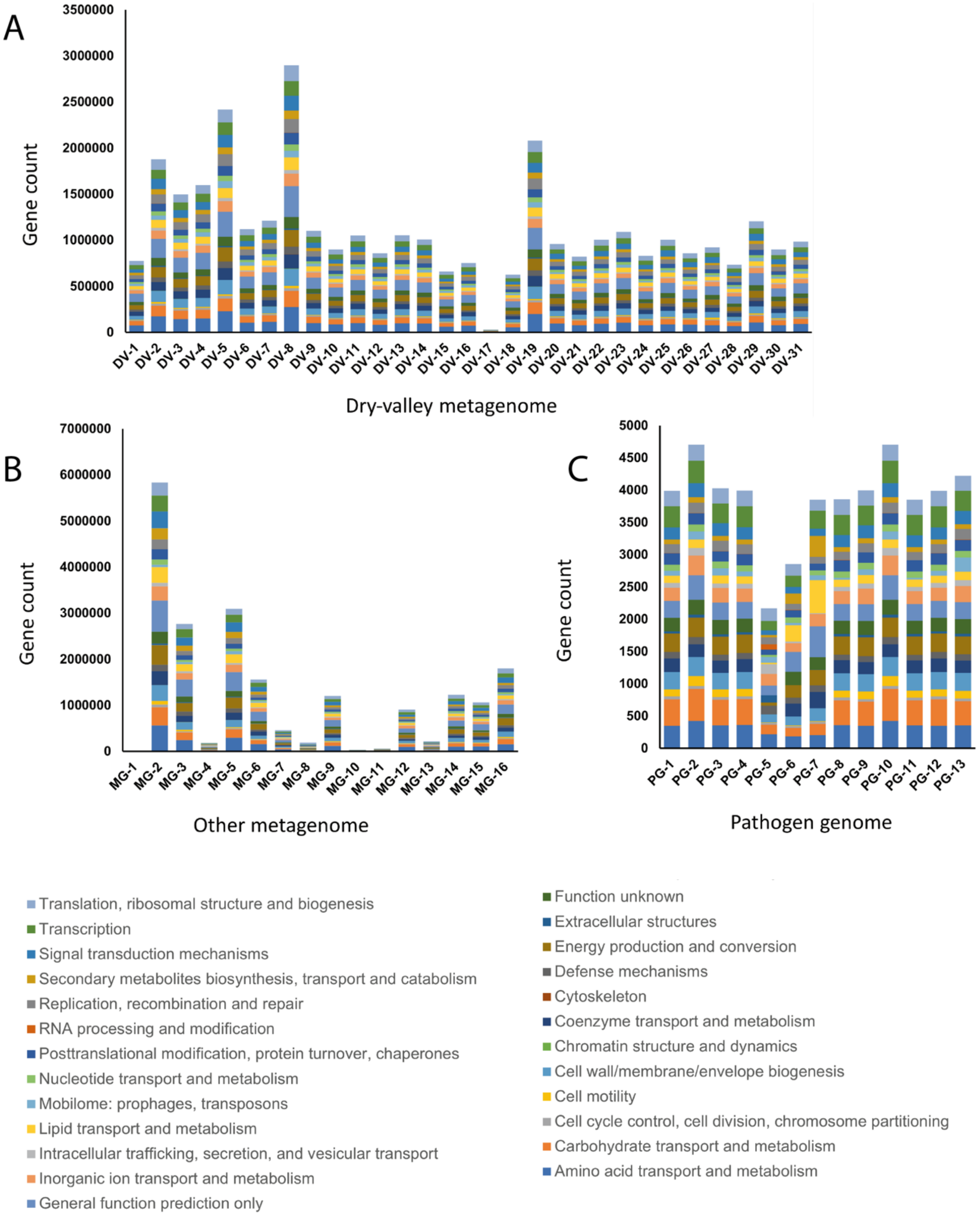

Supplementary 10. Percentages of genes in each COG category. Counts of genes in each COG category. GOLD identifiers and descriotion of each sample is given in in Supplementary 1

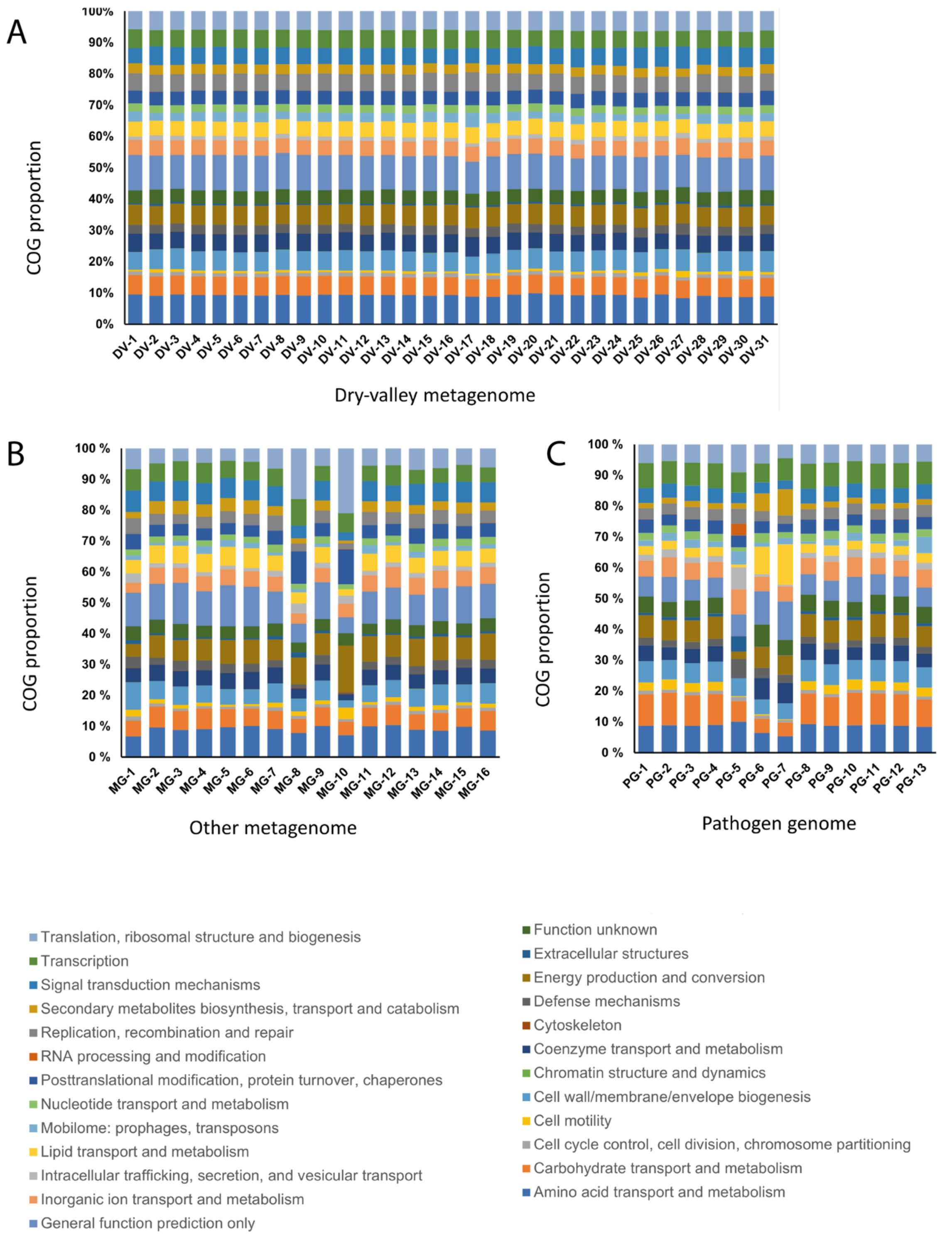

Supplementary 11. Sequence similarity networks built from sequences retrieved with searches from the MutS profile (PF01624). A) . Sequences with prediction for MutS alone. Dry-Valley metagenome nodes are coloured blue, UniRef50 nodes are indicated in red. B) Sequences including MutS_II, MutS_III, and MutS_V domains in addition to the MutS prediction.

A

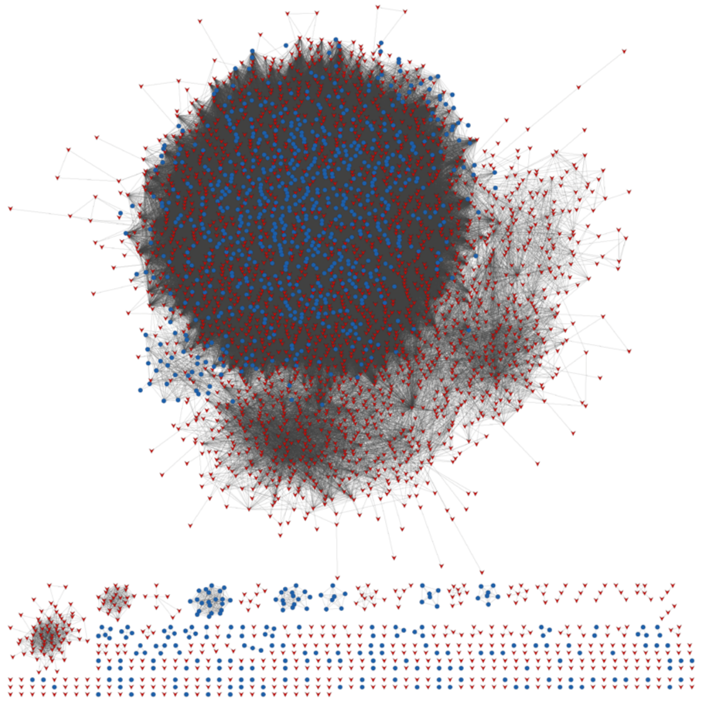

B

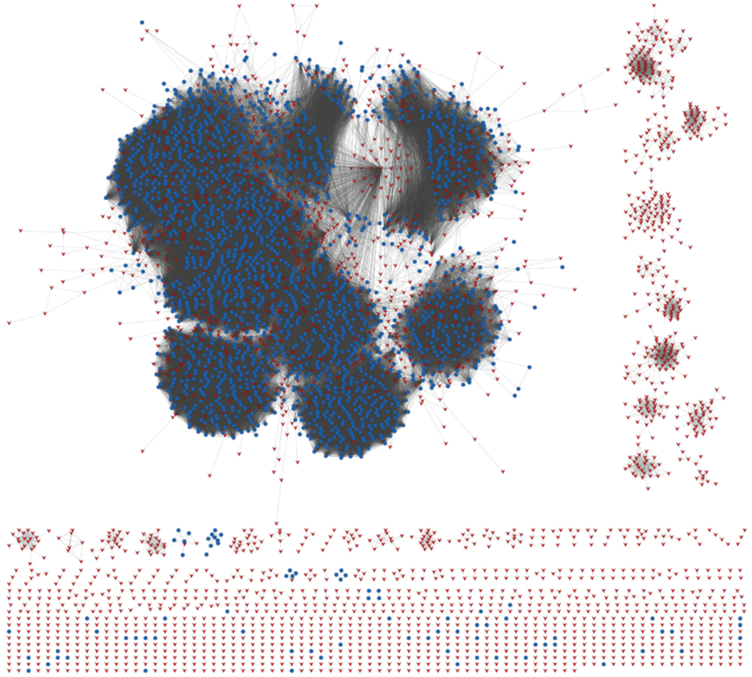

Supplementary 12. A) SSN of metagenome hits to the UvsE Pfam at the 54% identity threshold; other network parameters are detailed in Table 2. Dry-Valley metagenome nodes are colored blue, UniRef50 nodes are indicated in red. Cluster numbers discussed in the text are given in parentheses. B) Alignment of selected full-length sequences from metagenome clusters 1-3 to UvsE from D. radiodurans (UVES_DEIRA).

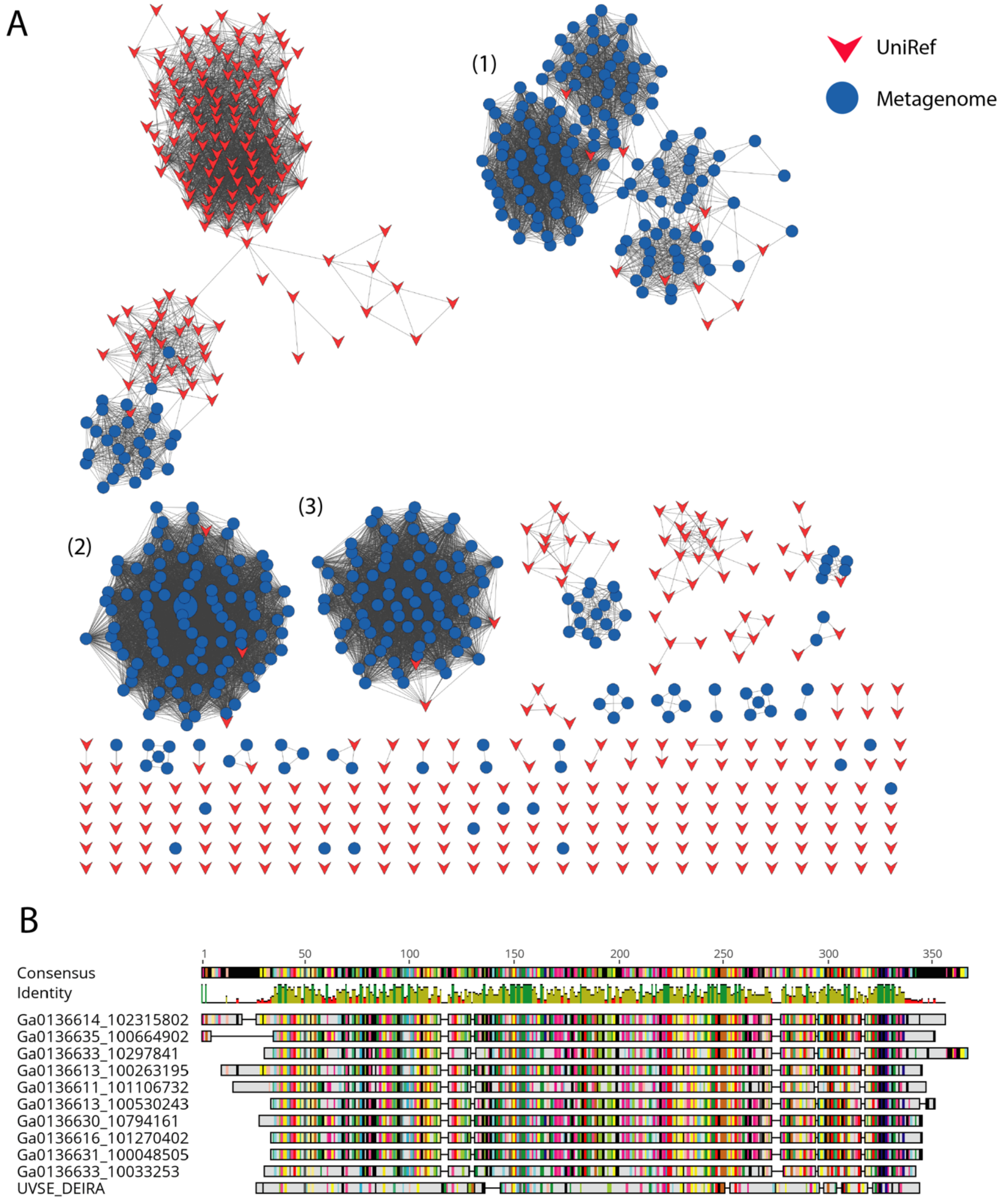

Supplementary 13. SSN of metagenome hits to the Rad21/Rad22 family. At the 50% identity edge threshold. Dry-Valley metagenome nodes are colored blue, UniRef50 nodes are indicated in red. The sub-clusters which derive from Cluster #1 at the 26% edge threshold (Figure 5) are indicated as 1a and 1b.

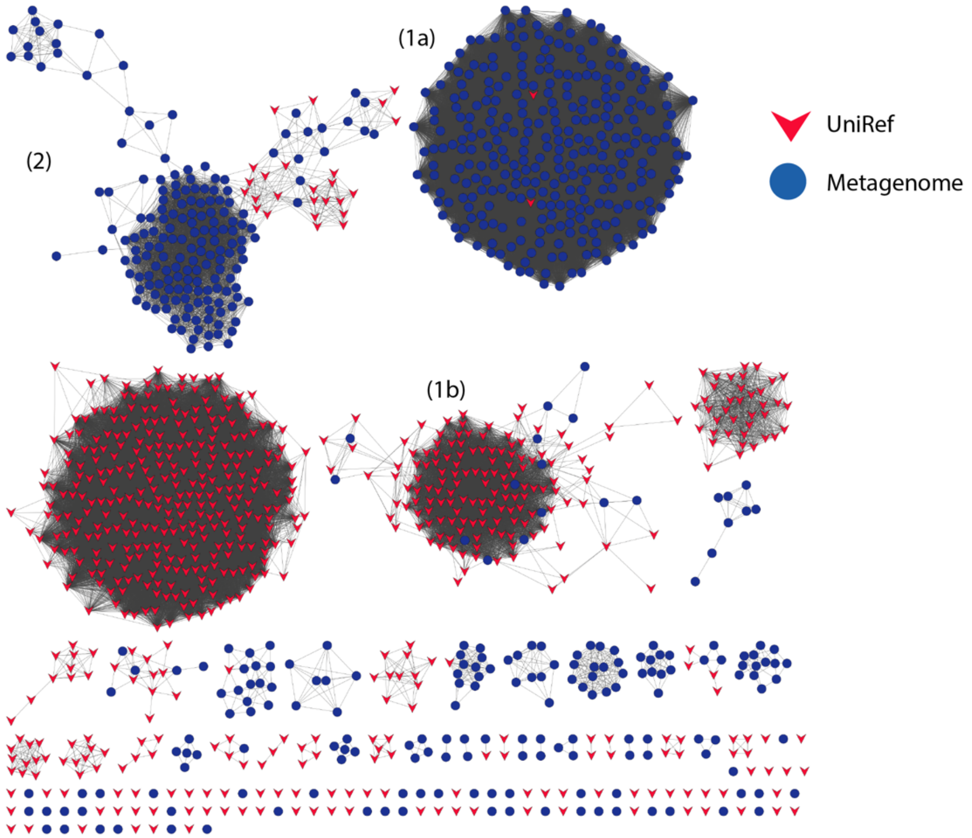

Supplementary 14. A) SSN of metagenome hits to the DdrB Pfam with 25% identity edge threshold. Dry-Valley metagenome nodes are colored blue, UniPro nodes are indicated in red. Cluster numbers discussed in the text are given in parentheses. B) Alignment of protein sequences from each cluster, including truncated and partial sequences, with DdrB from D. radiondurans (DEIRA_DdrB)

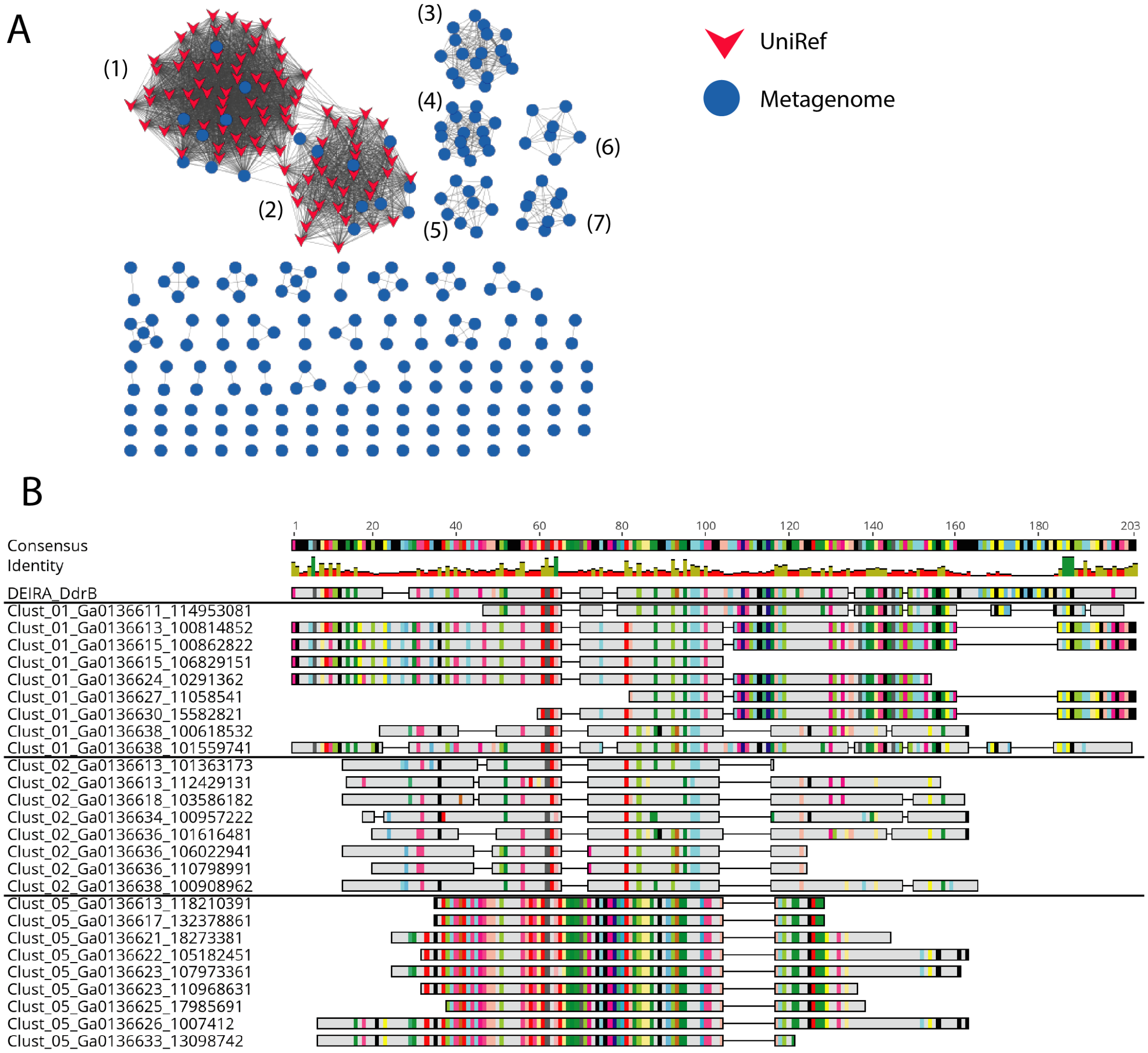

Supplementary 15. SSN of metagenome hits to LigB-type DNA ligases at 50% identity edge threshold. Domain compositions include the catalytic DNA_ligase_A_M domain together with the N-terminal DNA binding domain DNA_ligase_A_N. Nodes are colored by sequence length. The sequence used in further analysis is indicated by a red circle.

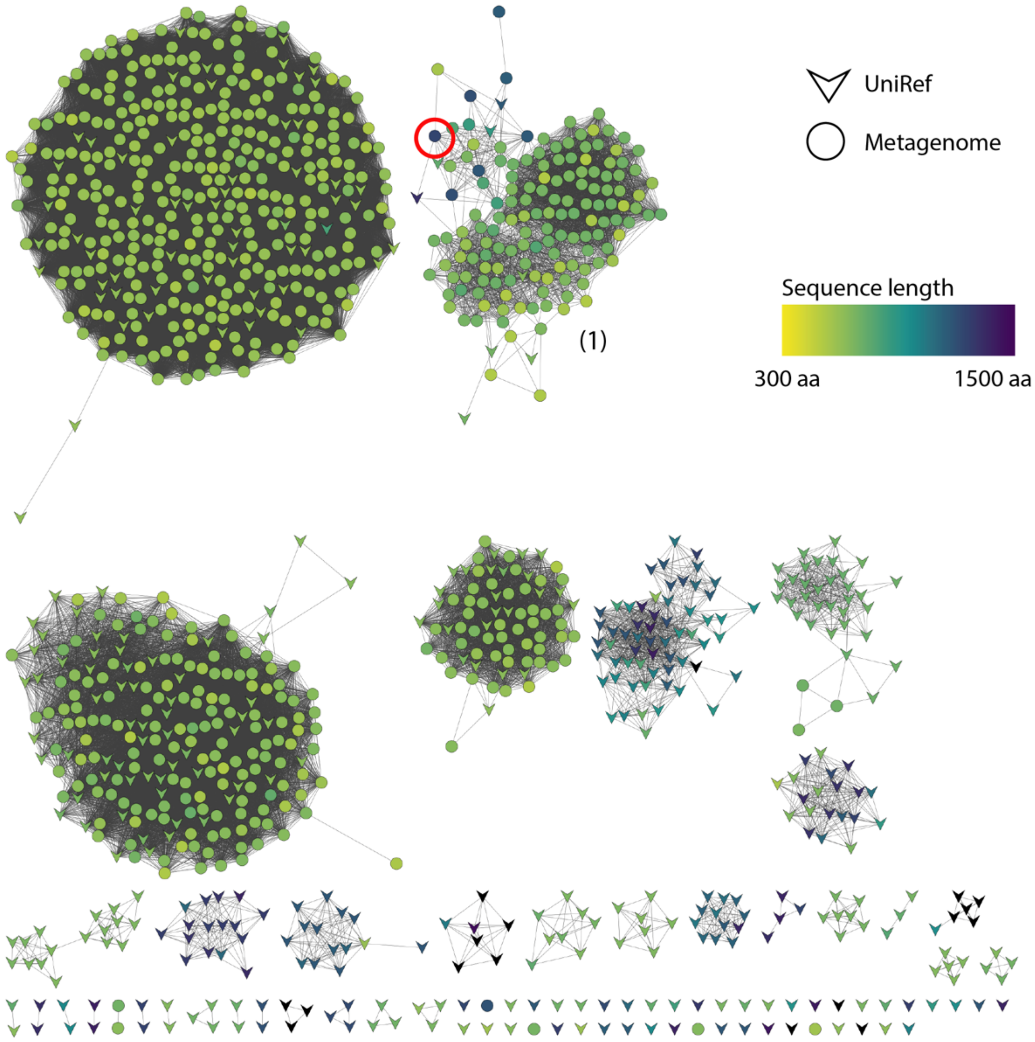

Supplementary 16. Annotations of genes adjacent to RMMBL nuclease-ligase (PF04675, PF07521) shown in Figure 6. Annotations for Dry Valleys genes are taken from IMG; annotations for *Opitutus terrae* are taken from the whole genome sequence NC_010571.

**Ga0136611_10000860**	Gene number in figure
Hypothetical protein	
Glycosyltransferase	1
Trehalose	2
23S rRNA methyltransferase	3
Small subunit ribosomal protein S9	4
Large subunit ribosomal protein L13	5
16S rRNA-methyltransferase	6
Molecular chaperone DnaJ	7
Molecular chaperone GrpE	8
Sigma-B regulation protein RsbU (phosphoserine phosphatase)	9
Anti-anti-sigma regulatory factor (antagonist of anti-sigma factor)	10
Hypothetical protein	
DNA ligase-1	
RecA DNA recombination protein	11
Protein ImuB	12
Error-prone DNA polymerase (DnaE-like)	13
Sugar phosphate isomerase/epimerase/AP endonuclease	14
**Ga0183260_10007327**	Gene number in figure
Sigma-B regulation protein RsbU	1
Antagonist of anti-sigma factor	2
Hypothetical protein	
DNA ligase-1	
Hypothetical protein	
Hypothetical protein	
Acyltransferase	3
Cytidylate kinase	4
**Ga0136615_10003237**	
Sigma-B regulation protein RsbU	1
Anti-anti-sigma regulatory factor	2
Hypothetical protein	
DNA ligase-1	
Hypothetical protein	
Hypothetical protein	
1-acyl-sn-glycerol-3-phosphate acyltransferase	3
Cytidylate kinase	4
**Ga0136636_10002227**	Gene number in figure
UDP-3-O-[3-hydroxymyristoyl] glucosamine N-acyltransferase	1
Outer membrane protein	2
Outer membrane protein insertion porin family	3
Large subunit ribosomal protein L9	4
Single-strand DNA-binding protein	5
Small subunit ribosomal protein S6	6
Peptidyl-tRNA hydrolase	7
Large subunit ribosomal protein L25	8
DNA ligase I	
Ribose-phosphate pyrophosphokinase	9
**Ga0136639_10000286**	Gene number in figure

Hypothetical protein			
DNA-binding transcriptional regulator YiaG	1		
Hypothetical protein			
Adenosylmethionine-8-amino-7-oxononanoate aminotransferase	2		
Hypothetical protein			
Membrane protease YdiL, CAAX protease family	3		
Predicted amidophosphoribosyltransferases	4		
Acetyl-CoA carboxylase carboxyl transferase subunit beta	5		
Dihydrofolate synthase	6		
DNA ligase I			
Signal peptidase I	7		
Phytoene synthase	8		
Hypothetical protein			
Menaquinone-specific isochorismate synthase	9		
REP element-mobilizing transposase RayT	10		
**NC_010571_Opitutus_terrae_PB90-1**		Locus tag	Protein ID
ABC transporter permease CDS	1	OTER_RS15970	WP_012375964.1
YdeI/OmpD-associated family protein CDS	2	OTER_RS15965	WP_012375963.1
PadR family transcriptional regulator CDS	3	OTER_RS15960	WP_202795987.1
Hypothetical protein		OTER_RS15955	WP_148218149.1
Serine hydrolase CDS	4	OTER_RS15950	WP_237702371.1
Hypothetical protein CDS		OTER_RS15945	WP_012375959.1
AI-2E family transporter CDS	5	OTER_RS15940	WP_012375958.1
Zinc ribbon domain-containing protein CDS	6	OTER_RS25330	WP_083767863.1
ATP-dependent DNA ligase CDS		OTER_RS15935	WP_012375957.1
DEAD/DEAH box helicase CDS	7	OTER_RS15930	WP_012375956.1
Metallophosphoesterase CDS	8	OTER_RS24300	WP_012375955.1
DUF3108 domain-containing protein CDS	9	OTER_RS15920	WP_012375954.1
DNA-3-methyladenine glycosylase CDS	10	OTER_RS15915	WP_012375953.1
PfkB family carbohydrate kinase CDS	11	OTER_RS15910	WP_012375952.1
Hypothetical protein		OTER_RS15905	WP_012375951.1
RplU 50s ribosomal subunit L21	12	OTER_RS15900	WP_012375950.1
RpmA 50s ribosomal subunit L27	13	OTER_RS15895	WP_012375949.1
Hypothetical protein		OTER_RS15890	WP_012375948.1
Hypothetical protein		OTER_RS15885	WP_044891812.1
Rod shape-determining protein CDS	14	OTER_RS15880	WP_012375946.1

Supplementary 17. SSN of metagenome hits to LigD-type DNA ligases with 54% identity edge threshold. Domain compositions include the DNA_ligase_A_M together with either the PrimaseS and/or the LigD_N domain Pfams. UniRef50 nodes are indicated in red. Cluster numbers discussed in the text are given in parentheses.

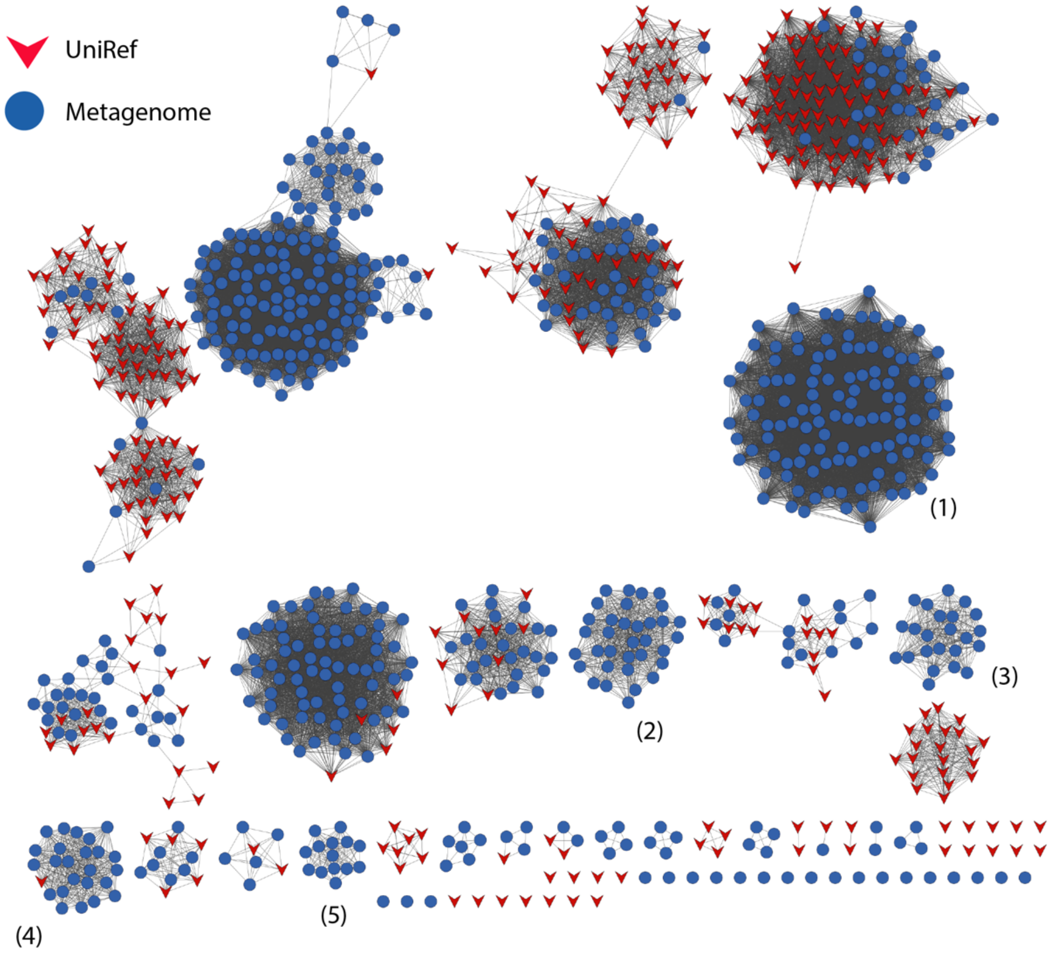

Supplementary 18. SSN of metagenome hits to DNA ligases with the catalytic DNA_ligase_A_M domain, and excluding the LigB and LigD appending domains. UniRef50 nodes are indicated in red. Cluster numbers discussed in the text are given in parentheses.

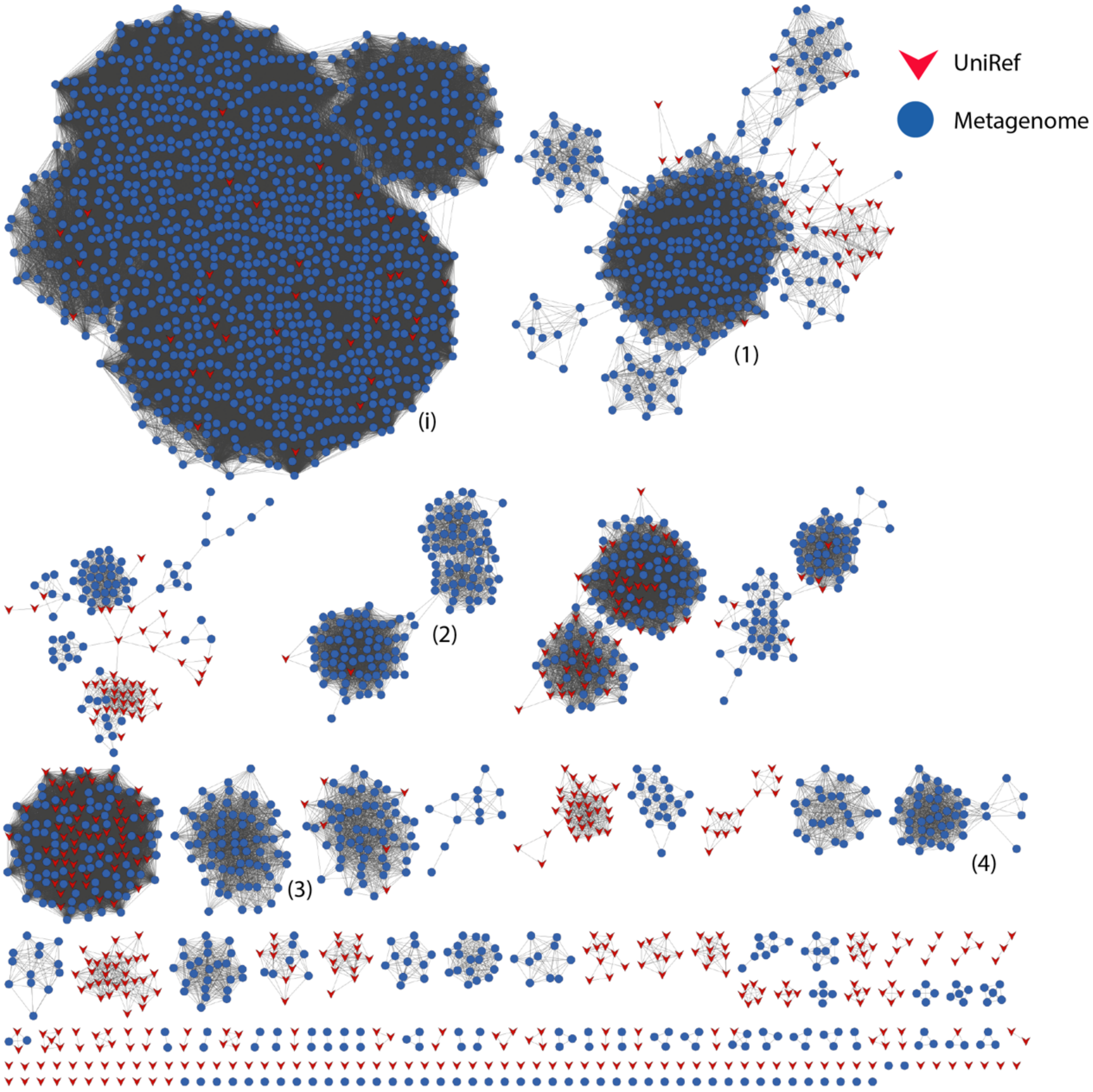

Supplementary 19. SSN of metagenome hits to DNA ligases with the HhH-GPD domain at the 25% edge threshold. UniRef50 nodes are indicated in red. Cluster numbers discussed in the text are given in parentheses.

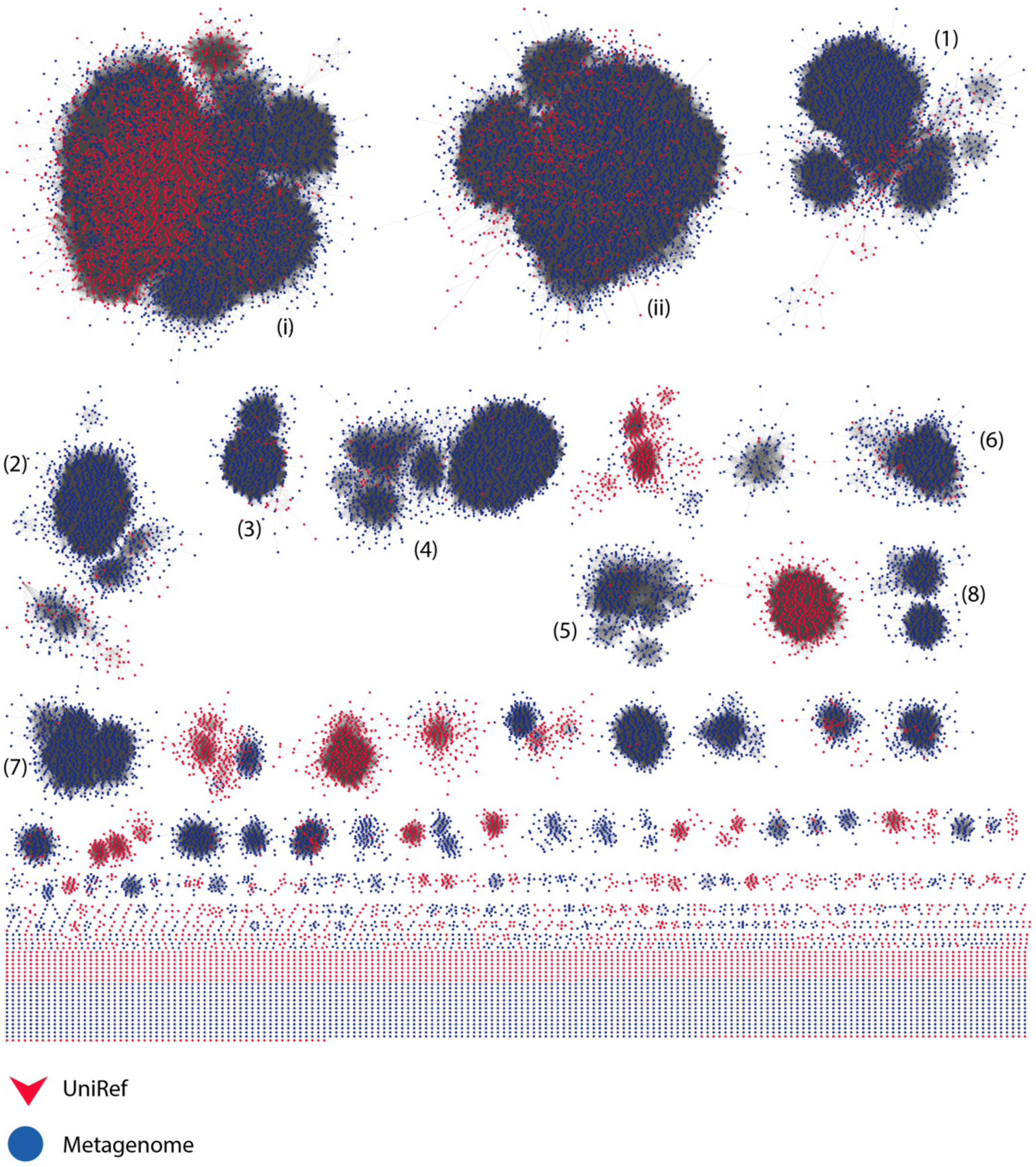

Supplementary 20. SSN of metagenome hits to DNA ligases with the HhH-GPD and AlkA_N domains at the 40% edge threshold. UniRef50 nodes are indicated in red. Cluster numbers discussed in the text are given in parentheses.

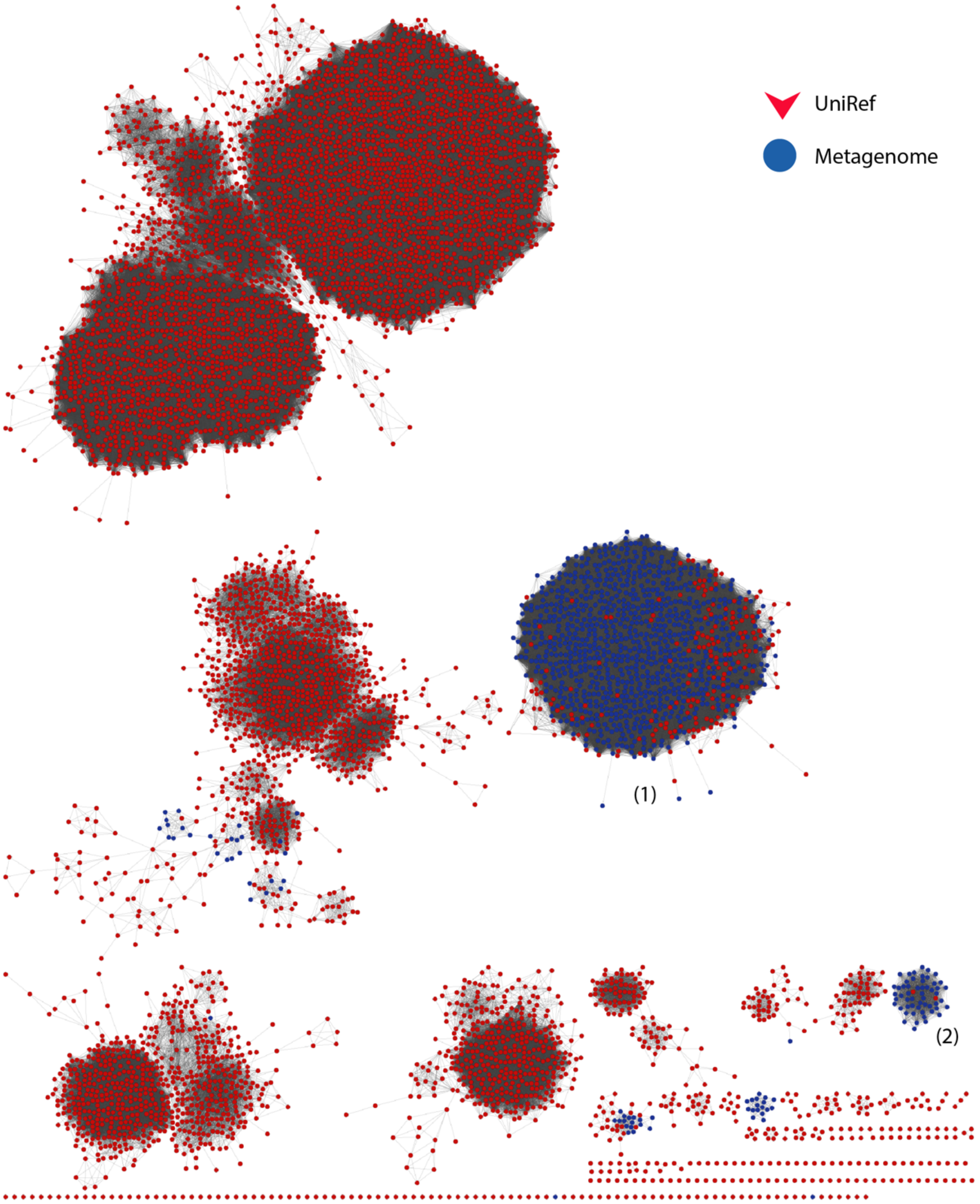

Supplementary 21. SSN of metagenome hits to DNA ligases with the DNA_photolyase domains at the 40% edge threshold. UniRef50 nodes are indicated in red. Cluster numbers discussed are given in parentheses.

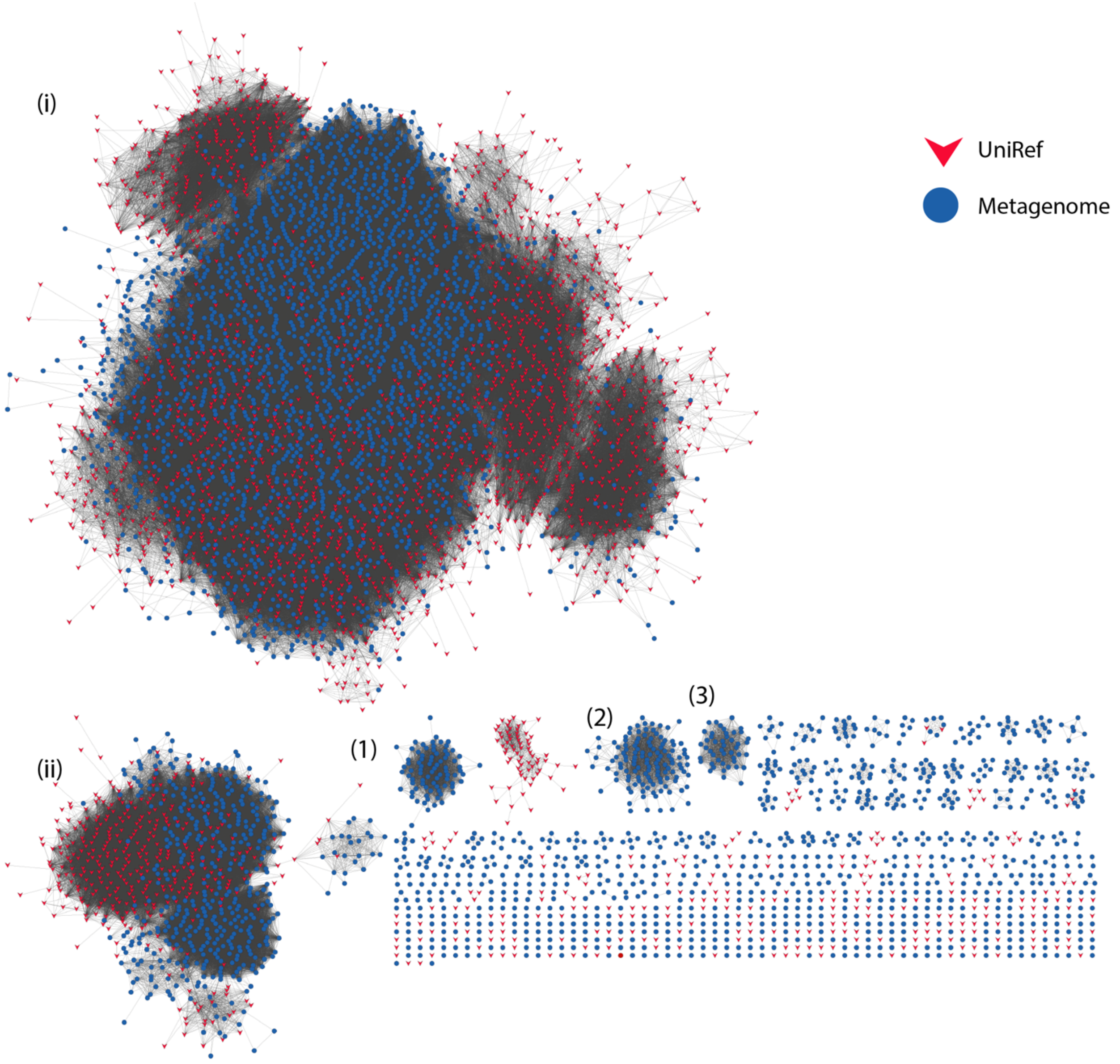

Supplementary 22. A. SDS PAGE gels for production of DV-NucS from E. coli BL21 pLysS. (i) Lanes 1-3 are insoluble (I), Crude extract (C) and soluble (S); lanes 4 – 14 are fractions eluted during the imidazole gradient in the first IMAC step, the red bar indicates fractions retained for the next step; lanes 15-17 are pooled desalted fractions from IMAC, pooled fractions with TEV at time 0 and after overnight incubation respectively. (ii) Flow-through fractions after reverse IMAC, red bar indicates fractious retained for the next step. (iii) Fractions from size exclusion. B. A. SDS PAGE gels for production of DV-Hjc from E. coli BL21 pLysS (i) Lanes 1-3 are crude lysate, soluble fraction and flow-through from the first IMAC. Fractions 4-12 are fractions eluted during the imidazole gradient in the first IMAC step, the red bar indicates fractions retained for the next step. (ii) TEV cleavage of pooled, desalted fractions at time 0 (1), after 30 min at room temperature (2) and after overnight at 4 °C at pH 8.0 (3) or pH 6.5 (4). Lanes 5 and 6 are fractions from reverse IMAC after cleavage. (iii) Fractions from size exclusion.

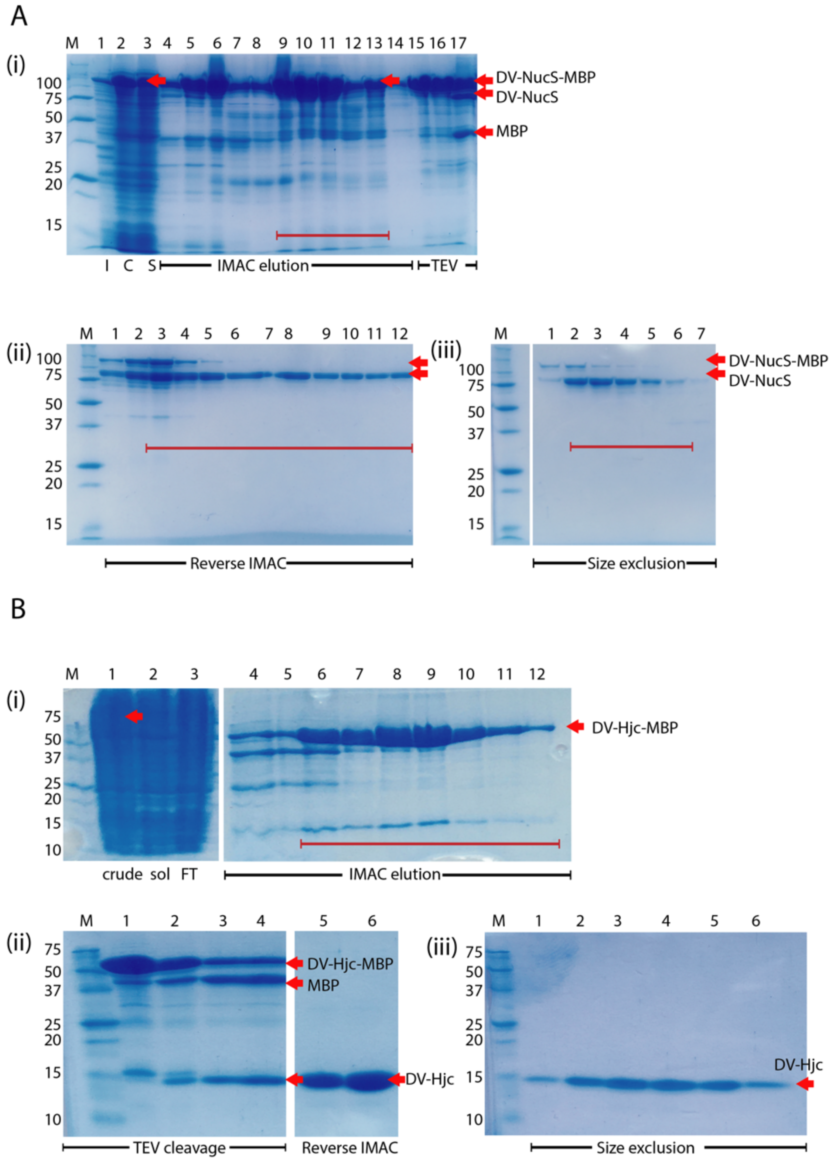

Supplementary 23. A) Expression of DV-Lig2 from (DE3) Origami (DE3). (i) Lanes 1-3 are; insoluble (I), flow though (F/T) and soluble (S); lanes 4-9 are fractions eluted during the imidazole gradient in the first IMAC step, the red bar indicates fractions retained for the next step. (ii) Lanes 1-2 are; pooled IMAC fractions before the addition of TEV and fractions after an overnight incubation with TEV; lanes 3-12 are flow-through fractions after reverse IMAC; lanes 13-14 are fractions eluted during the imidazole gradient in the reverse IMAC, red bar indicates fractions retained for the next step. (iii) Fractions from size exclusion, red bar indicates fractions that were kept for up-concentration. B) Production of DV-Lig5 from Origami (DE3). (i) Lanes 1-2 are insoluble (I), and soluble (S); lanes 3-9 are fractions eluted during the imidazole gradient in the first IMAC step, the red bar indicates fractions retained for the next step. (ii) Lanes 1-2 are pooled IMAC fractions before the addition of TEV (1) and fractions after overnight incubation with TEV (2); lanes 3-12 are flow-through fractions after reverse IMAC; lanes 13-14 are fractions eluted during the imidazole gradient in the reverse IMAC, red bar indicates fractions retained for the next step. (iii) Fractions from size exclusion, red bar indicates fractions that were kept for up-concentration

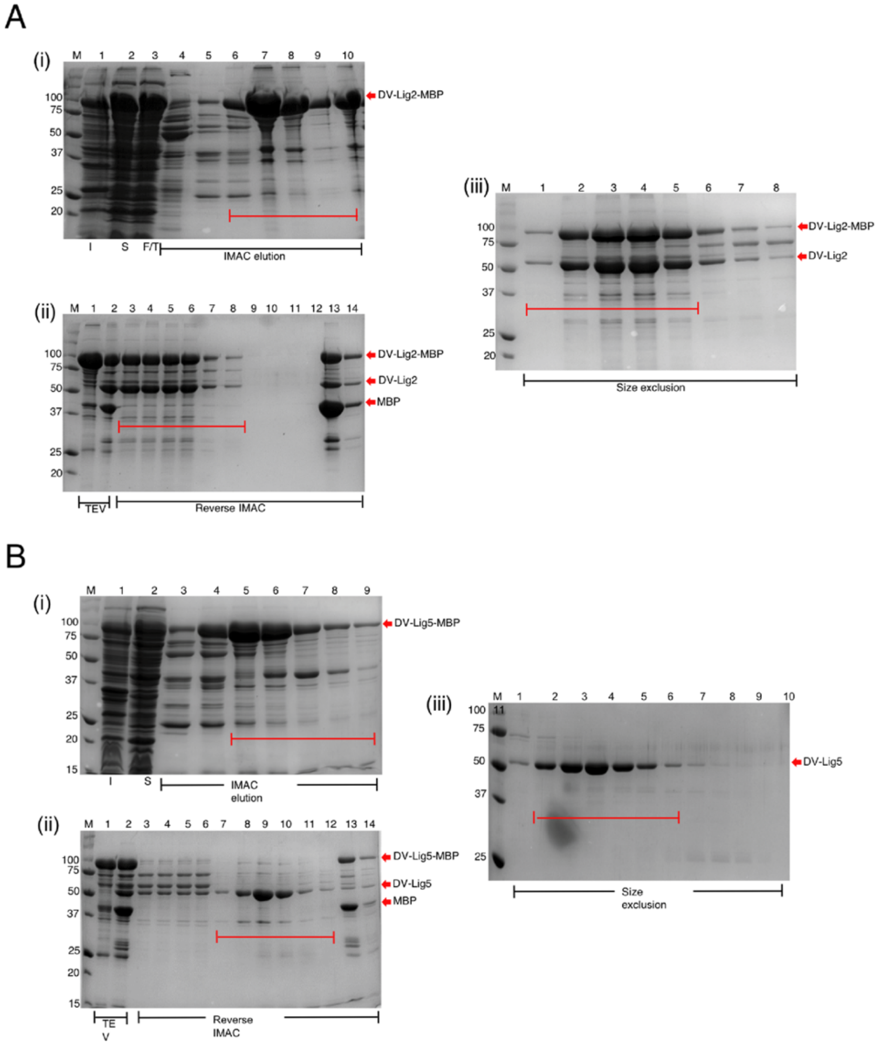

Supplementary 24. A) Schematic of DV-1-1-Nuc-Lig indicating boundaries and molecular weights of truncated expression constructs for individual enzyme annotations. B) SDS PAGE gels of of DV-1-1-Nuc expression trial showing soluble/ insoluble results. Lane 1 contains insoluble protein (P), lane 2 contains soluble protein (S) and lane 3 contains protein bound to Ni beads (N). The red box indicates the correct size of the nuclease which is only observed in lane 1 (P). C) SDS PAGE gels for production of DV1-1-Lig from E. coli (DE3) Origami. (i) Lanes 1 and 2 are insoluble (I), and soluble (S); lanes 3 – 9 are fractions eluted during the imidazole gradient in the first IMAC step, the red bar indicates fractions retained for the next step. (ii) Fractions from size exclusion.

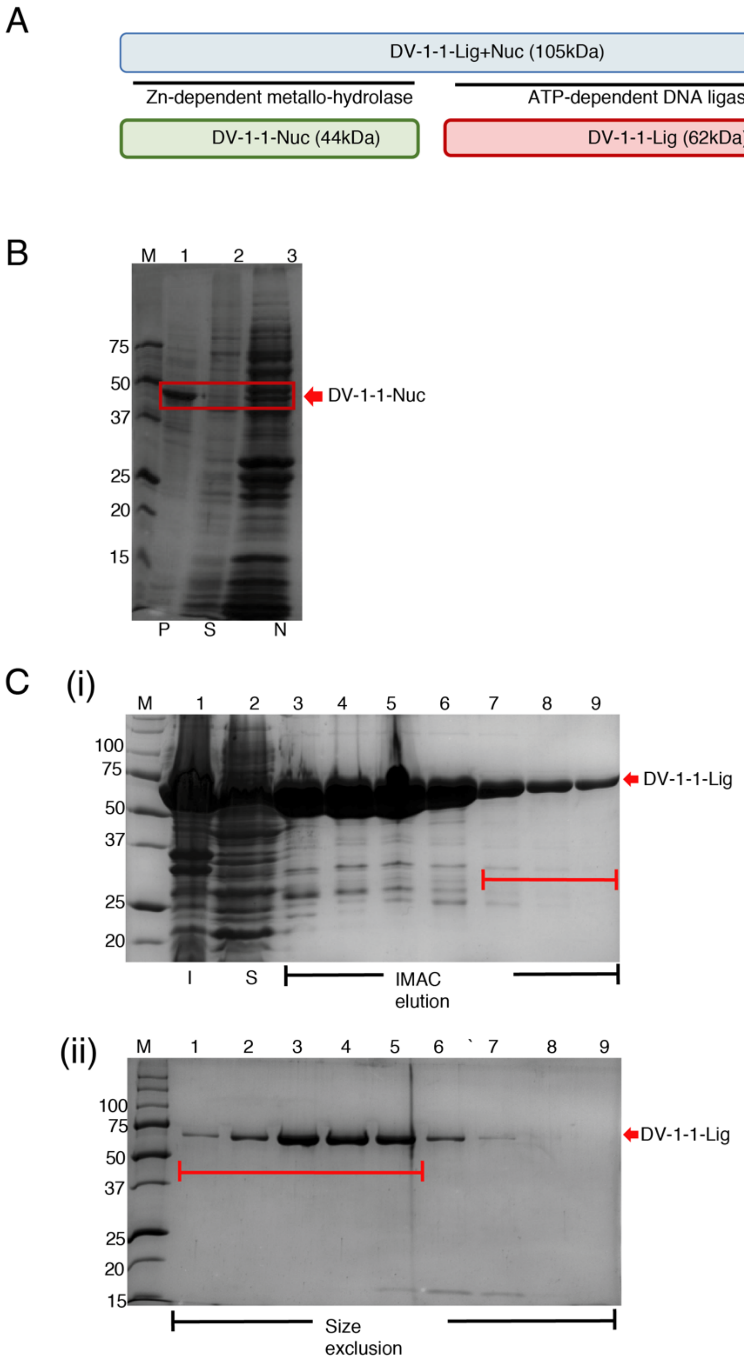

Supplementary 25. A) Nuclease assay of DV-Hjc on double-stranded DNA substrates containing damaged or mis-matched bases at a central position analyzed by denaturing urea PAGE. B) Nucleolytic activity of DV-Hjc on DNA substrates with double and single-stranded sections analyzed on 10% native TBE gels. C) Nuclease assay of DV-Hjc on DNA substrates containing flaps and junctions analyzed on denaturing urea PAGE. For all assays, substrates were incubated for 18 hours with 3.3 μM DV-Hjc (DV-Hjc gel) or an equivalent volume of buffer (control gel) at 25 °C. D) Thermal stability analysis o of DV-Hjc using differential scanning fluorimetry; (i) Normalized fluorescence signal change with temperature. (ii) Variation of Tm with pH.

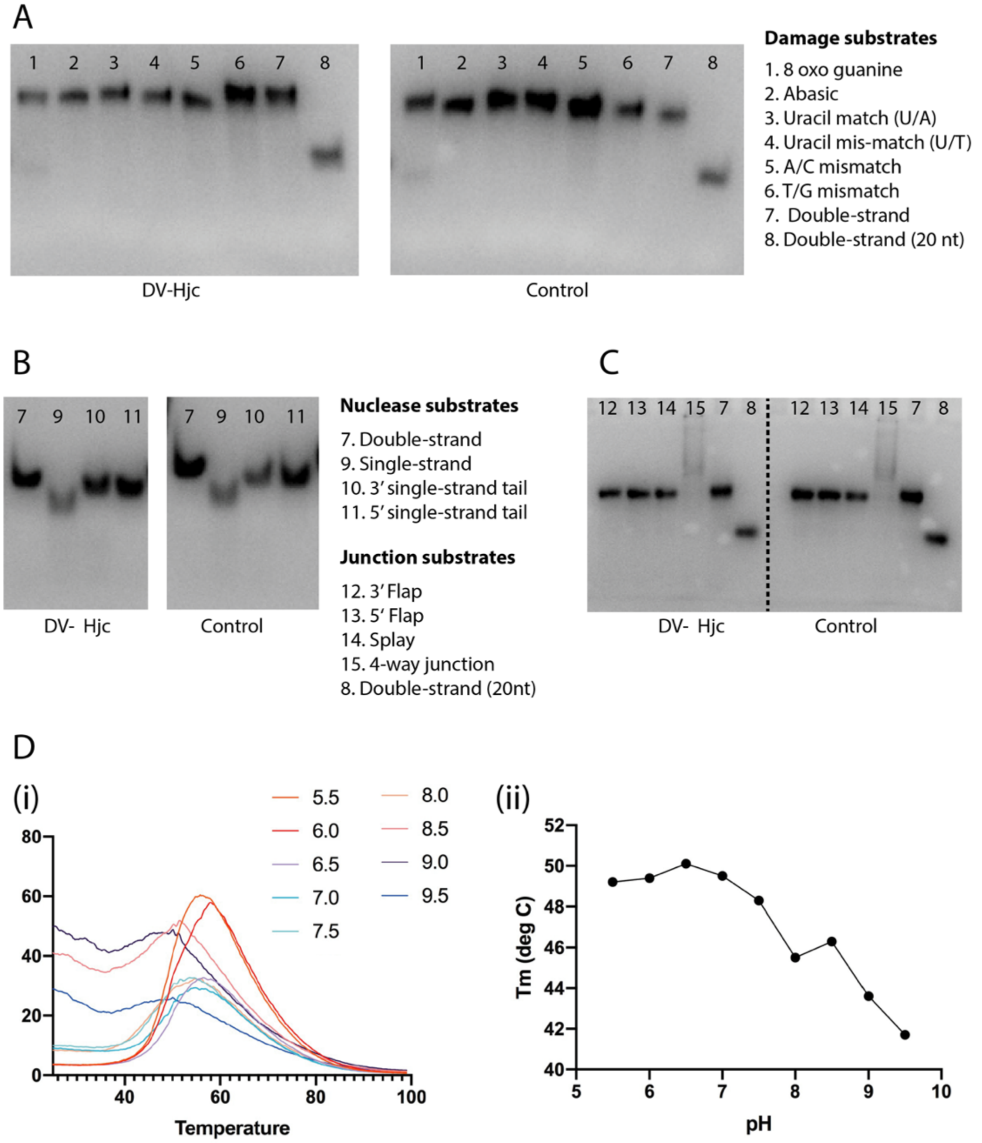

Supplementary 26. Organisation of large contigs in IMG. Predicted open reading frames are coloured by COG assignment and the gene if interest is indicated in red A) Contigs containing DV-NucS genes with the adjacent lysophospholipase labeled B) Contigs containing DV-Hjc genes with the adjacent RNAseHII labeled.

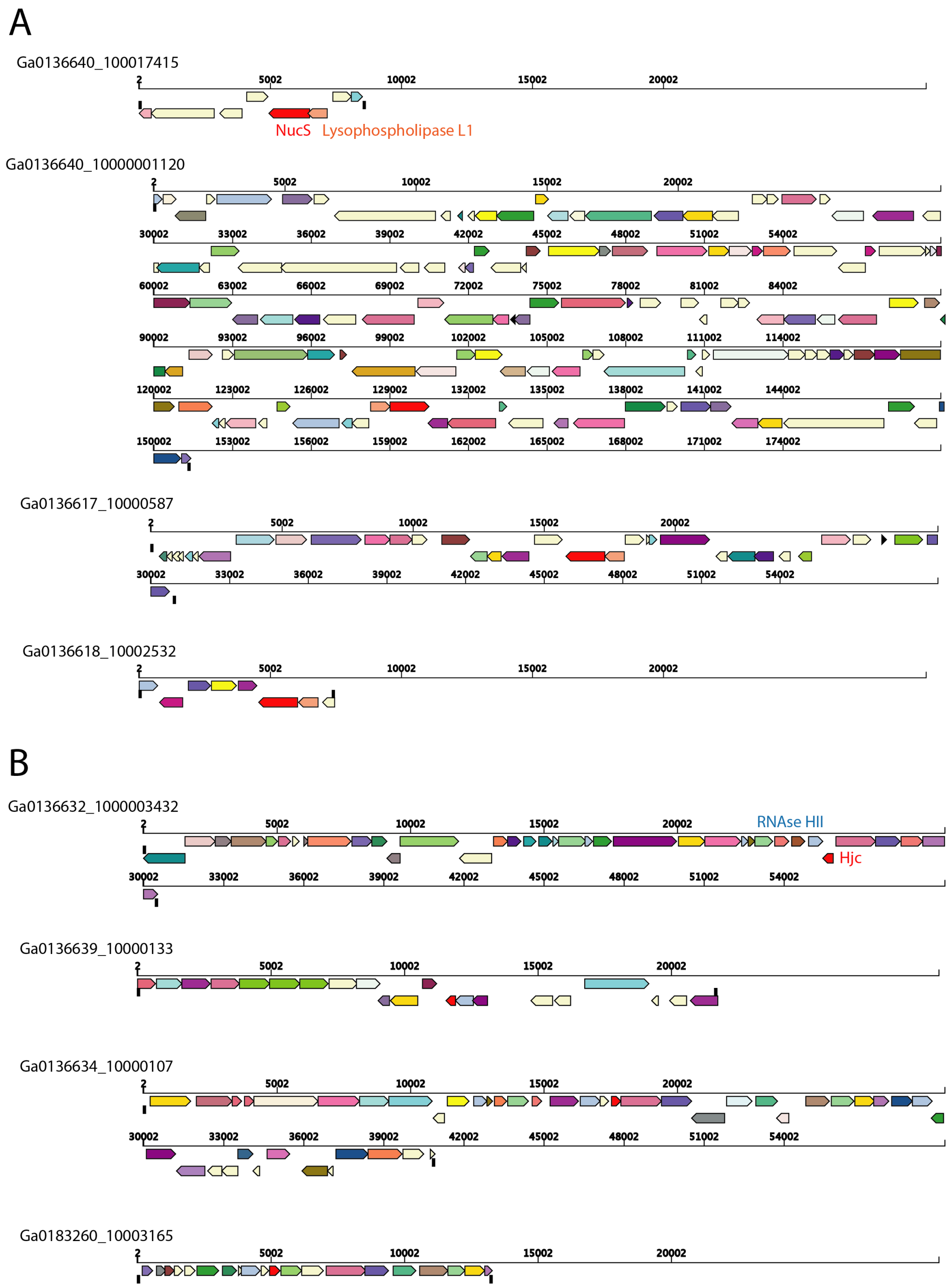

Supplement: Supplementary file 2 [file Data_Sheet_2.PDF]
